# Lab-scale characterization and semi-field trials of *Wolbachia* Strain *w*AlbB in a Taiwan *Wolbachia* introgressed *Ae. aegypti* strain

**DOI:** 10.1371/journal.pntd.0010084

**Published:** 2022-01-11

**Authors:** Wei-Liang Liu, Hui-Ying Yu, Yu-Xuan Chen, Bo-Yu Chen, Shiang Ning Leaw, Cheng-Han Lin, Matthew-P. Su, Ling-Shan Tsai, Yi Chen, Shin-Hong Shiao, Zhiyong Xi, Anna C-C Jang, Chun-Hong Chen

**Affiliations:** 1 National Mosquito-Borne Diseases Control Research Center, National Health Research Institutes, Miaoli, Taiwan; 2 Institute of Clinical Medicine, College of Medicine, National Cheng Kung University, Tainan, Taiwan; 3 School of Medicine, College of Medicine, I-Shou University, Kaohsiung, Taiwan; 4 Institute for Advanced Research, Nagoya University, Nagoya, Japan; 5 Department of Biological Science, Nagoya University, Nagoya, Japan; 6 Public Health Bureau, Tainan City Government, Tainan, Taiwan; 7 Chang Bing Show Chwan Memorial Hospital, Changhua, Taiwan; 8 Department of Tropical Medicine and Parasitology, College of Medicine, National Taiwan University, Taipei, Taiwan; 9 Department of Microbiology and Molecular Genetics, Michigan State University, East Lansing, Michigan, United States of America; 10 Department of Biotechnology and Bioindustry Sciences, National Cheng Kung University, Tainan, Taiwan; 11 National Institute of Infectious Diseases and Vaccinology, National Health Research Institutes, Miaoli, Taiwan; 12 Institutes of Molecular and Cellular Biology, National Taiwan University, Taipei, Taiwan; Connecticut Agricultural Experiment Station, UNITED STATES

## Abstract

Dengue fever is one of the most severe viral diseases transmitted by *Aedes* mosquitoes, with traditional approaches of disease control proving insufficient to prevent significant disease burden. Release of *Wolbachia*-transinfected mosquitoes offers a promising alternative control methodologies; *Wolbachia*-transinfected female *Aedes aegypti* demonstrate reduced dengue virus transmission, whilst *Wolbachia*-transinfected males cause zygotic lethality when crossed with uninfected females, providing a method for suppressing mosquito populations. Although highly promising, the delicate nature of population control strategies and differences between local species populations means that controlled releases of *Wolbachia*-transinfected mosquitoes cannot be performed without extensive testing on specific local *Ae*. *aegypti* populations. In order to investigate the potential for using *Wolbachia* to suppress local *Ae*. *aegypti* populations in Taiwan, we performed lab-based and semi-field fitness trials. We first transinfected the *Wolbachia* strain *w*AlbB into a local *Ae*. *aegypti* population (*w*AlbB-Tw) and found no significant changes in lifespan, fecundity and fertility when compared to controls. In the laboratory, we found that as the proportion of released male mosquitoes carrying *Wolbachia* was increased, population suppression could reach up to 100%. Equivalent experiments in semi-field experiments found suppression rates of up to 70%. The release of different ratios of wAlbB-Tw males in the semi-field system provided an estimate of the optimal size of male releases. Our results indicate that *w*AlbB-Tw has significant potential for use in vector control strategies aimed at *Ae*. *aegypti* population suppression in Taiwan. Open field release trials are now necessary to confirm that *w*AlbB-Tw mediated suppression is feasible in natural environments.

## Author summary

Prior to open field release, new genetic approaches that interfere with mosquito abilities and reduce mosquito population density require progressive evaluation both in the laboratory and contained field trials. Trials in contained outdoor systems are thus an important step and allow for evaluation of the effectiveness and reliability of *Wolbachia*-infected *Aedes aegypti* (*w*AlbB-Tw) in settings that incorporate natural environmental variations. We describe a semi-field system designed to study the ability of *w*AlbB-Tw mosquitoes to suppress local mosquito populations. We provide a protocol for establishing, maintaining, and monitoring *Ae*. *aegypti* population densities inside field cages. These results pave the way for the open release of *w*AlbB-Tw-infected *Ae*. *aegypti* mosquitoes in the field.

## Introduction

Dengue virus (DENV) is a member of the Flaviviridae family which has four serotypes (denoted DENV-1 to DENV-4) and causes dengue fever [1]. Every year around 400 million people worldwide are infected with DENV, making it one of the largest public health threats in tropical and subtropical countries [2,3]. DENV is mainly transmitted by *Aedes* (*Ae*.) *aegypti* and *Ae*. *albopictus*, and widely used and highly safe vaccines and antiviral treatments currently exist [2]. In addition to interventions targeting the disease via the vector, such as insecticide application, clinical management can also be used to intervene in the spread of the disease [4]. However, existing strategies have proven insufficient for dealing with the significant increase in cases of dengue fever reported worldwide [5]. The significant dengue fever outbreaks in southern Taiwan, in 2014 and 2015 resulted in hundreds of deaths. Although there have been no serious outbreaks of dengue fever in this region since then, sporadic cases still occur. Therefore, new strategies to combat dengue (and *Aedes*-borne diseases in general) that can be applied in Taiwan specifically are urgently required.

*Wolbachia* is an intracellular symbiont found mainly in arthropods [6–8]. Mosquito species naturally infected with *Wolbachia* include *Culex pipiens* and *Ae*. *albopictus*, but not *Ae*. *aegypti* [9,10]. Recent studies have shown that infection with specific *Wolbachia* strains can inhibit a variety of human pathogens in mosquitoes, including dengue, chikungunya, yellow fever, West Nile and Zika viruses, as well as the malaria parasite [11–15]. *Wolbachia* presents within the cell cytoplasm and is consequently transmitted vertically from infected females to their offspring. *Wolbachia* can manipulate host population via cytoplasmic incompatibility (CI), and this regulatory ability not only exists in transinfected insects but also in natural infections. When *Wolbachia*-transinfected males mate with females that are uninfected or harboring a different *Wolbachia* type, early embryo death follows [16,17]. As uninfected males can successfully mate with infected females, with their offspring developing normally, *Wolbachia*-transinfected females thus gain a reproductive advantage which allows the endosymbiotic bacterium to invade and persist in populations. *Wolbachia*-induced CI has been proposed as a method to produce functionally infertile males as part of a population suppression strategy to reduce the size of wild populations in the next generation. This strategy has been successfully implemented in many agriculturally important insects and is considered to be cost-effective for insect population control. It is also considered an environmentally friendly method that has no impact on human health and has been proven to be highly sustainable. A CI based strategy is currently being used to successfully control mosquito populations in many countries worldwide, with field releases of male *Aedes* mosquitoes carrying *Wolbachia* in the United States, Australia, China, Singapore and Italy significantly reducing wild *Aedes* mosquito densities [18–21]. Though this is promising, there are still local differences between *Ae*. *aegypti* populations, so any promising studies in other countries will need to be replicated again in Taiwan before any attempts at local population suppression are made.

*Wolbachia* can also protect its hosts against a wide range of pathogens, with the extent of this protection dependent on strain type [22,23]. For instance, the *w*Mel *Wolbachia* strain has strong anti-DENV properties and few host fitness costs in *Ae*. *aegypti*. Moreover, this strain can also successfully invade *Ae*. *aegypti* populations [24,25]. In addition, the *w*MelPop strain could be stably introduced into *Ae*. *aegypti* to indirectly reduce DENV transmission by reducing mosquito lifespan [26,27]. It has also been found that different *Wolbachia* strains in *Ae*. *albopictus* also have different biological functions in the interspecific transfer. For instance, the *w*Pip strain does not induce viral blocking and does induce complete CI; alternatively, although *w*Au induces very strong virus blocking (likely much stronger than *w*Mel) it does not induce CI [28,29]. Recent reports suggest that *w*AlbB shows almost perfect maternal transmission and CI, even in high-temperature environments [30,31], and can reduce or block replication of a wide range of Flavivirus and Alphavirus species under laboratory conditions [32]. Further, *w*AlbB can effectively suppress mosquito population and block DENV transmission to humans in the field [33–35]. *w*AlbB’s ability to induce almost complete CI lends strong support to the idea of using this strain as part of a population suppression strategy. Successes with this strategy include eliminating *Cx*. *pipiens* in a village in Myanmar and a recent effort to control *Ae*. *polynesiensis* [18,36]. In addition, application of a combination of CI and sterile insect technology in China and Thailand has significantly reduced the density of *Ae*. *albopictus* and *Ae*. *aegypti* in their pilot study areas [18,37]. Google Verily’s Debug Project, which focuses on the release of *Wolbachia*-infected male mosquitoes, has shown a 95.5% reduction in wild adult mosquito populations across three replicate release sites in Fresno County, California [19]. All of these strategies however rely on *w*AlbB-infected males remaining competitive relative to wild type males in order to facilitate local suppression or eradication efforts.

Several factors have played an important role in the success of the large-scale implementation of *Wolbachia’s* suppression program. Among these, the ability for laboratory mass-reared and sterilized males to compete with wild males for wild female mating success is critical. Laboratory tests for competitiveness include evaluating laid eggs, lifespan, size, sperm production, or mating ability [38–40]. These factors can be affected by every step in the mosquito production process. Therefore, formulating a standard protocol for mosquito release is necessary. Many experiments have been performed under laboratory and semi-field conditions to study the effect of the release ratio on inducing CI [18–20,32,37]. These studies highlight that male mating competitiveness is hugely important for achieving effective population suppression. Because so many different factors can change the effectiveness of a population suppression program, their successful deployment in a local environment (such as Taiwan) requires the detailed study of these infected mosquitos, their life cycle, and their suppression effectiveness in both laboratory and small-scale field settings that mimic local conditions.

We herein conducted an in-depth study of *Wolbachia* infection of local *Ae*. *Aegypti* and studied its potential for deployment. We first transinfected native Taiwanese *Ae*. *aegypti* with *Wolbachia* and verified the biological function of the constructed *w*AlbB-Tw, including vector competence and male mating competitiveness in both lab and semi-field tests. We secondly studied the ability of *w*AlbB-Tw males to suppress local mosquito populations in different release ratios. Through this series of research and data collection, we obtained the basic information necessary for the implementation of *Wolbachia* prevention technology in Taiwan. These studies will be crucial in advancing to the next stage of field trials for the release of *w*AlbB-infected males.

## Materials and methods

### Ethics statement

The mouse blood used in this study was purchased directly from BioLASCO Taiwan Co., Ltd. (http://www.biolasco.com.tw). The company acts in accordance with the Guide for the Care and Use of Laboratory Animals, regulations on live vertebral animal experiments, and ARRIVE guidelines.

### Mosquitoes

We used two different strains of *Ae*. *aegypti* for experiments: a local Tainan city field strain (WT) and a *Wolbachia-*transinfected mosquito (*w*AlbB-Tw) propagated through a series of backcrosses with a WT genetic background. The WT strain was established from larvae collected from residential premises across Tainan during routine inspections by Taiwan National Mosquito-Borne Diseases Control Research Center (NHRI) enforcement officers. The WT strain was established by collecting and hatching eggs from an ovitrap placed in the field in Tainan City. This line was then reared and expanded for 5 generations. Mosquitoes were reared at 28°C ± 1°C and 70% ± 10% relative humidity under 12 h light/12 h dark conditions. Parental mosquitoes were allowed to mate randomly and were fed with commercially obtained aseptic breeding mouse’s blood using an artificial membrane blood feeder system (Orinno Technology Pte. Ltd., Singapore). Mosquito eggs were allowed to hatch after two days in water. We reared 4,500 larvae in plastic containers measuring 40 × 60 × 8 cm^3^, each containing approximately 4 L of water, and fed them a mixture of yeast (Taiwan Sugar Corporation, Taiwan) and goose liver powder (#7573, NTN Corporation, Taiwan) in a 1:1 ratio. Pupae were placed inside 30 x 30 x 30 cm^3^ transparent acrylic cages prior to emergence as adults. Adults were provided with a constant supply of 10% sucrose solution.

### Generating a local *Wolbachia-*transinfected *Ae*. *aegypti* strain (*w*AlbB-Tw)

To generate a local *Wolbachia*-transinfected *Ae*. *aegypti* strain (*w*AlbB-Tw), 200 female *w*AlbB-infected *Ae*. *aegypti* from the WB2 line obtained from Michigan State University [41] were backcrossed with equal numbers of local WT male *Ae*. *aegypti*. In the following generation, 200 hybrid females were mated with 200 WT males. This backcrossing was repeated for ten generations following the rearing methods for WT mosquitoes described above. In addition, both male and female mosquitoes in every generation were assayed for *Wolbachia* infection using polymerase chain reaction (PCR) tests. The specific primers for *w*AlbB were as follows [42]: forward 5′-ACGTTGGTGGTGCAACATTTG-3′; reverse 5′- TAACGAGCACCAGCATAAAGC-3′. At least ten individuals from each established line were sequenced. This outcrossing was repeated for ten generations to create stable genetic backgrounds for subsequent experiments and semi-field release.

### *Wolbachia* visualization in the midgut and ovary

Dissected midguts and ovary tissue from females (about five days old) were fixed and permeabilized using a fixative buffer (4% paraformaldehyde, 0.1 M HEPES, and 1% Triton X-100 in PBS) for 30 min. Samples were then washed five times with 0.1% PBS-Triton X-100 before being blocked with 3% BSA (bovine serum albumin) for 2 h at room temperature. Blocked midguts were stained with rabbit polyclonal *Wolbachia* antibody (1:500 dilution) overnight at 4°C [43]. After three washes with TBST (Tris-buffered saline with 0.1% Tween 20), the midguts were incubated for 2 h at room temperature with Alexa Fluor 488 goat anti-rabbit IgG-labeled antibody in TBST (1:500 dilution). After several more washes in TBST, midguts were stained with 1 μg/mL DAPI and phalloidin (Abcam, Cambridge, UK) for 10 min. After mounting with mounting gel, tissues were imaged using a Leica TCS SP5 confocal microscope (Leica, Wetzlar, Germany).

### Cytoplasmic incompatibility (CI) assay

The CI level was measured by the cage population method, which was based on the studies of Zabalou et al. [44]. All of the crosses were performed at 28°C. The crossing tests were as follow: WT male x WT female, *w*AlbB-Tw male x WT female, WT male x *w*AlbB-Tw female, and *w*AlbB-TW male x *w*AlbB-Tw female. Twenty virgin females (three days old) were mated with 20 virgin males (three days old), with three replicates completed.

Mated females were fed blood using an artificial membrane blood feeder system. The study lasted for two weeks and blood was fed once a week so that the female mosquitoes could lay eggs at least 2 times during the experimental period. Two egg papers were collected from each cage. Oviposition sites were constantly available to females, and oviposition paper was changed weekly. After five days of egg maturation on wet filter paper, eggs were immersed in deoxygenated water that had been evacuated by vacuum. Three days later, hatched eggs were counted to determine hatch rates.

### DENV maintenance

DENV serotypes 1–4 were propagated in Vero cells. Briefly, Vero cells, grown in a 175 mm^2^ flask at a confluency of approximately 80% at 28°C, were infected at a multiplicity of infection of 0.5 for 3 h at 28°C. Later, fresh medium was added and the infection proceeded for 5 days at 28°C. Finally, the cell culture supernatant was filtered through a 0.22-μm filter, aliquoted, and stored at −80°C until use.

### Mosquito infection with DENV and virus titration

Fresh mouse blood was centrifuged at 4°C for 10 min to separate plasma and blood cell components. The plasma was then heat treated at 55°C for 1 h. Blood cells were washed three times with PBS, after which plasma and blood cell components were mixed. Virus supernatant was combined with treated mice blood to yield 2 × 10^7^ PFU/mL virus blood. This infected blood was then provided to mosquitoes via an artificial membrane feeder for 30 min. Successfully blood-fed mosquitoes were visually identified and maintained separately from those that were not blood-fed. Saliva of these infected mosquitoes was collected accordingly [45]. Briefly, the legs and wings of infected mosquitoes were removed, and their mouthparts inserted into a P200 tip which was filled with 5 μL of FBS. 30 min later, the FBS contained in the tip was added to 45 μL of serum-free medium in RPMI and then tested via plaque assay.

### Plaque assay

To determine DENV-2 titer, 2 x 10^5^ BHK21 cells were seeded into individual wells of a six-well plate and incubated for 24 h. Virus supernatant was serially diluted (10^−1^–10^−7^) with DMEM, then applied to BHK21 cells in the six-well plate. After infection for 2 h, virus supernatant was removed and cells were washed with PBS. Cells were then covered with DMEM containing 1% methyl cellulose and 2% FBS, then incubated for another five days. After incubation, the DMEM was removed and cells were fixed and stained with 0.5% crystal violet [46]. Plaque forming units (PFU) were then calculated to determine viral titers.

### Insecticide susceptibility assay

The conducted space spray experiments used previously published protocols for quantifying mosquito knockdown and mortality [47]. Females from the three mosquito strains were tested: the local wild strain of *Ae*. *aegypti* (WT), *w*AlbB-Tw, and the laboratory-generated *Ae*. *aegypti* (LG), which originated from a field population collected in the municipality of Kaohsiung. The laboratory-generated strain has been maintained in the Department of Entomology, National Chung Hsing University, Taiwan since 2004. All the tests in this study were performed with the 210th generation of this strain, with no resistance to drugs. Three insecticides commonly used in Taiwan were diluted to two different concentrations for insecticide susceptibility testing; deltamethrin was diluted to 960 and 60 ppm, α-cypermethrin was diluted to 4,400 and 275 ppm, and cyfluthrin was diluted to 2,040 and 127.5 ppm. A cylindrical nylon mesh bioassay cage (approximately 10 × 5 cm^2^ in diameter) was placed 30 cm above the ground, with each cage containing 25 *Ae*. *aegypti* from one of the tested mosquito strains. Thirty minutes prior to insecticide exposure, bioassay cages were placed in a 3.1 × 2.3 × 3 m^3^ room with no air circulation. Insecticide was applied by a technician wearing appropriate personal protective equipment (gloves and a mask) at a horizontal angle for 10 s. This procedure for the space spray formulation was done for a total of three replicates. For each replicate, knockdown was scored at 30 min using the absence of flight as the knockdown criterion, although this also included mosquitoes that had died. Mortality was then assessed for all mosquitoes 24 h post-spray.

### Female reproduction capacity assay

To estimate the impact of *w*AlbB presence on female fertility, assays were performed using 5- to 8-d old sex-separated mosquitoes. The virgin WT and *w*AlbB-Tw females were transferred into their respective acrylic cages by aspiration. Next, 60 WT males were put into 60 WT female cages, and 60 *w*AlbB-Tw males were put into 60 *w*AlbB-Tw female cages to allow for mating to occur. Females were fed mouse blood using an artificial membrane blood feeder system 3 d later, and non-blood sucking females and all males were removed from the cage. Three days after blood feeding, 40 female mosquitoes were anesthetized on ice for 5 minutes, and then transferred individually into plastic vials for egg collection. Each plastic vial contained 3 mL water and 3 × 2 cm^2^ filter paper. Three days later, the number of laid eggs, hatched eggs, larvae, and surviving adults were counted. Females that died during this time were excluded from the study.

### Mating capability test in cages

To determine mating capability, the following crosses were prepared: ♀ *w*AlbB-TW x ♂*w*AlbB-TW, ♀*w*AlbB-TW x ♂WT, ♀WT x ♂*w*AlbB-TW, and ♀WT x ♂WT. For each group, we pooled 5 males with 20 virgin females in transparent acrylic cages (30 × 30 × 30 cm^3^) under standard rearing conditions. After 1 h, female spermathecae were dissected and examined under a microscope for sperm insemination as an indicator of mating activity. Insemination rates or mating frequency was calculated as the percentage of inseminated females divided by the number of dissected females. Each experiment was repeated three times.

### Lifespan assays

For laboratory and semi-field experiments investigating lifespan, we transferred 20 male or female mosquitoes (three days old) into 30 × 30 × 30 cm^3^ transparent acrylic cages and provided them with unlimited 10% sucrose solution. Female mosquitos were given a single blood meal 3 days after eclosion. For semi-field experiments, temperature, humidity, and sunlight hours were regulated by nature; the temperature fluctuated between 26 and 32°C with a relative humidity of approximately 80~95%, and about 14 hours of sunshine per day (April to June). For both types of experiments, dead mosquitoes were removed every day and recorded until no viable mosquitoes were left. Each experiment was repeated three times.

### Laboratory male mating competitiveness assay

To determine the mating competitiveness of *Wolbachia*-transinfected *Ae*. *aegypti* males, we transferred varying ratios of WT virgin females (♀WT), WT males (♂WT), and *w*AlbB-Tw male (♂*w*AlbB-Tw) (1:1:0, 1:1:1, 1:1:3, 1:1:5, 1:1:10) to either transparent acrylic cages or insect rearing tents (BugDorm-2960, 160 x 160 x 180 cm^3^), with five replicates performed for each experiment. We pooled 10 ♂WT and 0, 10, 30, 50, or 100 ♂*w*AlbB-Tw in each individual cage. Before introducing 10 virgin ♀WTs, the two types of males were first mixed together for 2 hours. Mosquitoes were allowed to mate for two days and then blood fed for 30 min. Two days after blood feeding, egg cups were inserted into the transparent acrylic cages or insect rearing tents for egg collection. The number of eggs laid and the hatching rate of larvae were counted by collecting the egg cups. After five days of egg maturation on wet filter paper, eggs were immersed in deoxygenated water. Three days later, hatched eggs were counted to determine hatch rates.

### Suppression of wild type populations by release of *w*AlbB-Tw males under semi-field conditions

We constructed a natural semi-field environment to analyze the ability of *Wolbachia* to suppress mosquito populations. The semi-field area was approximately 80 m^3^ (3.8 m × 9.4 m × 2.3 m^3^) and located in Tainan city, Taiwan (22.956395, 120.196156). The building was located in well-ventilated farmland surrounded by two layers of 150 mesh white nylon mesh to prevent mosquitoes from escaping and other insects from entering, with the outermost layer covered with 100 mesh black nylon mesh to protect all semi-field buildings from UV rays. Inside the semi-field, four wooden cabin structures simulating houses were situated at each experimental site to mimic a place for a shelter, blood feeding, and laying eggs. The bottom of each cabin leg was placed into cups filled with water in order to prevent ants from reaching sucrose solution contained in plastic cups, which were placed on top of the table. The remainder of the internal site was covered with mulch and potted plants to emulate a typical yard. Sensors were located inside the test site to monitor ambient temperature and relative humidity.

Mosquitoes were transported from the mass-rearing factory to the release sites and were released between 3:00 ~ 5:00 p.m. Cotton soaked with 10% sucrose solution was continuously supplied to the adult mosquitoes before release. The mosquito release ratio followed the above mentioned ratio tested in the laboratory. We pooled 200 ♂ WT and 600, 1000, or 2000 ♂ *w*AlbB-Tw in each individual cage. Before introducing 200 virgin ♀ WTs, the two types of males were first mixed together for 2 hours. In the semi-field test, we still used the artificial membrane blood feeder system to feed the mated females. Two days after release, the blood feeder system in the wooden cabin was initiated, the blood plate bag was replaced every 2 hours, and the feed was provided for 10 hours a day (8:00 a.m. ~ 6:00 p.m.) for 2 days. These experiments were conducted for more than ten months across three seasons, and mosquitoes were released on average once a week for population suppression experiments. The number of eggs laid and the hatching rate of larvae were counted by collections from the ovitraps in the 4 wooden cabins for each experiment. After five days of egg maturation on wet filter paper, eggs were immersed in deoxygenated water. Three days later, hatched eggs were counted to determine hatch rates.

### Data analysis

All statistical analyses were conducted using either the GraphPad Prism software, version 5.0, or R-4.1.0. The differences in viral titer between WT and *w*AlbB mosquitoes were analyzed using Mann-Whitney rank sum tests. Statistical analyses of lifespan were performed using the Log-Rank test via R-4.1.0 [48]. We compared egg hatching rates for the mating competitiveness assays using paired t-tests for both laboratory and semi-field populations. A *p*-value less than 0.05 was considered statistically significant.

## Results

### Generating *w*AlbB-transinfected *Ae*. *aegypti* with Taiwan genetic background (*w*AlbB-Tw) and characterization of the *w*AlbB-Tw line

To create the local Taiwanese strain of *Wolbachia*-transinfected *Ae*. *aegypti*, we backcrossed the Tainan local field strain of *Ae*. *aegypti* with the *Wolbachia-*transinfected strain. The presence of *Wolbachia* was monitored in each generation by specific PCR assays. After 10 consecutive generations of testing, the backcrossed strains (*w*AlbB-Tw) can be stably transfected to the next generation with a transinfection rate of 100%.

**Fig 1:**
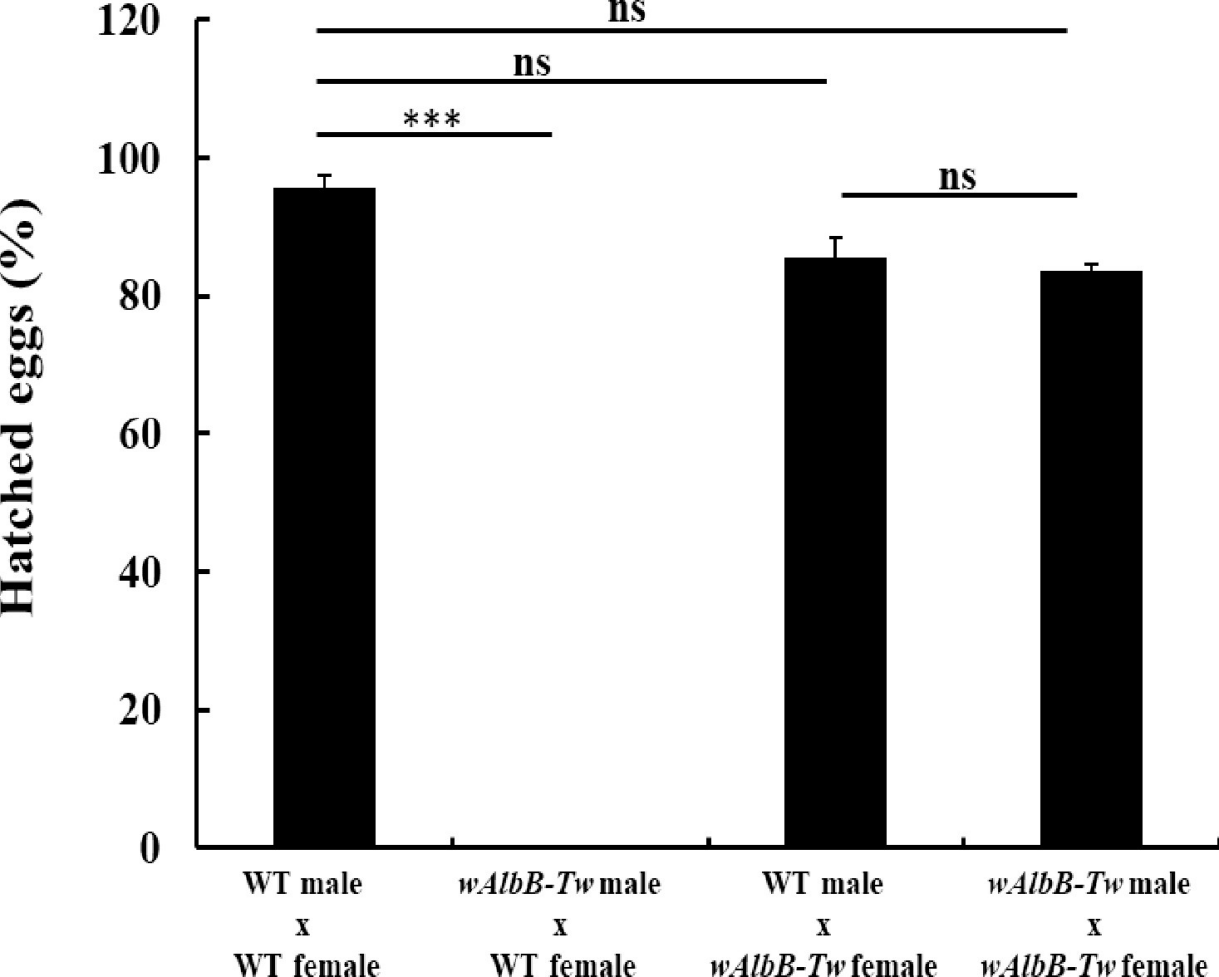
*Wolbachia* induced cytoplasmic incompatibility in *w*AlbB-Tw line. Egg hatch in reciprocal crosses between *w*AlbB-transinfected and wild-type *Ae*. *aegypti* lines (WT). The crossing tests were used as follow: WT male x WT female, *w*AlbB-Tw male x WT female, WT male x wAlbB-Tw female, and *w*AlbB-TW male x *w*AlbB-TW female. Data are represented as mean ± SD. Significance was determined using student t-test, ***p < 0.01, ns, not significant. WT: Tainan local wild type.

To further characterize the effect of CI in *w*AlbB-Tw lines, we used crossing experiments in transparent acrylic cages containing 20 uninfected WT females and equal numbers of *w*AlbB-Tw males from the transinfected line and found a hatching rate of 0% (Fig 1). Reciprocal crosses resulted in an 85.5±2.2% egg hatching rate under identical experimental conditions. Control crosses between WT or *w*AlbB-Tw males and females resulted in 95.6±1.4% and 83.6±0.9% egg hatching rates respectively (Fig 1). We also verified the anti-*Wolbachia* antiserum via immunofluorescence assay. As shown in S1 Fig, infected *w*AlbB-Tw mosquitoes show *Wolbachia* distribution in midgut and ovary tissue.

### DENV replication was reduced in *w*AlbB-Tw *Ae*. *aegypti*

To further verify that the newly generated *w*AlbB-Tw mosquito shows suppression of viral replication, one of the key characteristics of Wolbachia infection, virus suppression tests were performed. Mosquitoes were infected orally with DENV-1 to -4 and then incubated for 7- and 14- days post blood meal (PBM). After this the midguts, salivary glands and saliva of these mosquitoes were collected to test virus titers and infection rate (IR). The IR was defined the number of DENV-infected mosquitoes divided by the total number of mosquitoes. The viral titer per mosquito was measured via plaque assay.

Seven days after consuming a blood meal containing 2x10^7^ plaque-forming units (PFU/mL) of DENV, *w*AlbB-Tw females exhibited diverse anti-DENV capacity against all four serotypes (S2 Fig). In the WT sample, the median DENV-1 viral titer from the midgut was approximately 2.9 × 10^4^ PFU/mL, but in *w*AlbB-Tw the average titer was approximately 177 PFU/mL (*p* = 0.002, Mann-Whitney rank sum test). For DENV-2, the average viral titer in the control sample was approximately 6.8 × 10^4^ PFU/mL, compared with approximately 267 PFU/mL for the *w*AlbB-Tw line (*p* = 0.006, Mann-Whitney rank sum test). Similarly, *w*AlbB-Tw mosquitoes showed strong suppression of both DENV-3 and DENV-4 viruses (DENV-3; 227 PFU/mL vs. 1.3 × 10^4^ PFU/mL for the WT, *p* <0.01; DENV-4; 170 PFU/mL vs. 4.5× 10^4^ PFU/mL for the WT, *p* < 0.01). Following a longer incubation time of 14 days, we found that the viral titers of DENV-1 to DENV-4 in the *w*AlbB-Tw were also significantly lower compared to WT groups (S2 Fig and S1 Table).

Similar results were identified when comparing DENV viral titers in the salivary glands. For *w*AlbB-Tw, DENV-1 to DENV-4 viral titers were significantly lower both 7 and 14 dpi when compared to WT. Seven days PBM, WT mosquitoes showed clear evidence of DENV in their salivary glands, while fewer *w*AlbB-Tw individuals exhibited DENV infection (1.9 x 10^4^ PFU/mL vs. 69.1 PFU/mL for DENV-1; 4.2 x 10^4^ PFU/mL vs. 332.2 PFU/mL for DENV-2; 3.4 x 10^4^ PFU/mL vs. 649.3 PFU/mL for DENV-3; 3.6 x10^4^ PFU/mL vs. 484.2 PFU/mL for DENV-4). At 14 PBM, the *w*AlbB-Tw strain also showed antiviral effects (S2 Fig and S1 Table). After testing for potential suppressive effects in the salivary glands, we next tested viral titer in saliva. In the saliva, we once again found that *w*AlbB-Tw showed reduced viral titers. At 7 and 14 PBM, there was significant evidence of DENV-2 virus suppression in *w*AlbB-Tw as compared to WT (30 PFU/mL vs. 72 PFU/mL and 42 PFU/mL vs. 91 PFU/mL, respectively, *p* < 0.01; S2B Fig). Similar results were observed for DENV-1, DENV-3 and DENV-4, at 7 and 14 PBM, though these were not significant (S2A, S2C and S2D Fig and S1 Table).

Furthermore, *w*AlbB-Tw mosquitoes showed significantly reduced IR values compared with the control group for all DENV serotypes at day 7 PBM (;16.7% vs. 80% for DENV-1; 10% vs. 86.7% for DENV-2; 13.3% vs. 66.7% for DENV-3; 16.7% vs. 73.3% for DENV-4; Mann-Whitney test, *p* < 0.01; S2 Fig), as well as at day 14 PBM (S2 Fig).

### Insecticide susceptibility

Insecticide-based control is one of the most common strategies used to suppress *Ae*. *aegypti* populations in disease-endemic areas. Therefore, we assessed the impact of insecticides used on newly generated *Wolbachia* positive mosquitoes. We tested the difference in response to insecticides such as deltamethrin, alpha-cypermethrin and cyfluthrin between *w*AlbB-Tw and the uninfected strain (WT).

**Fig 2:**
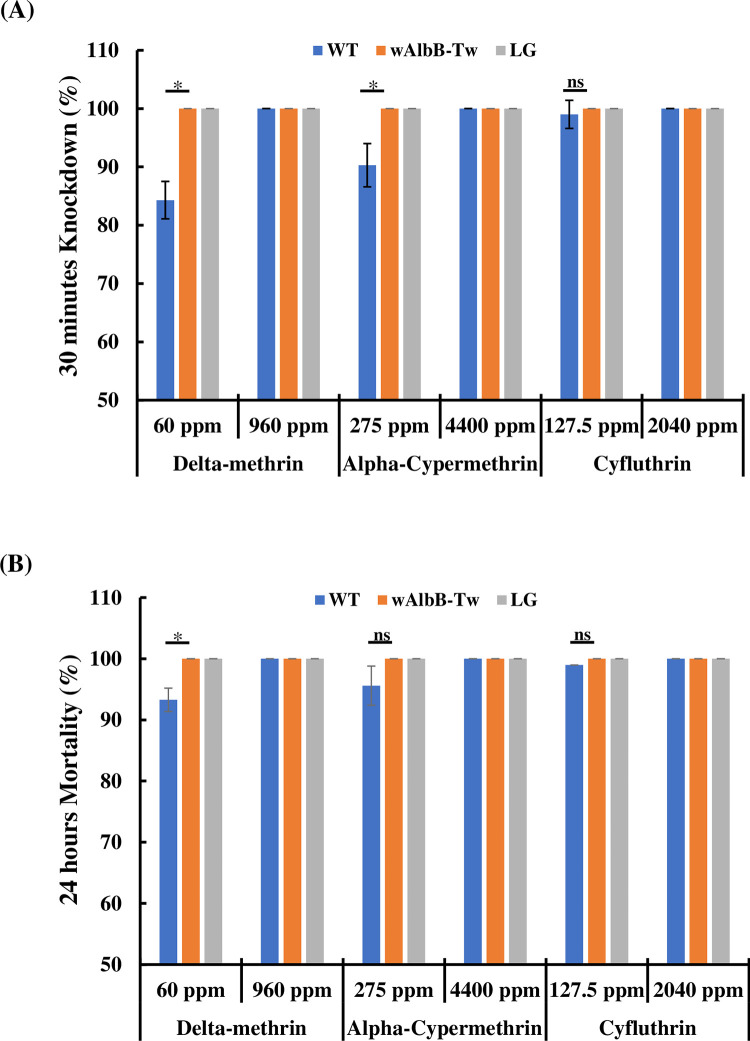
Mosquito susceptibility to three insecticides. Females from three mosquito strains were knocked down and their mortality recorded in a space spray trial assay. (**A**) Knockdown was scored at 30 min using the absence of flight as the knockdown criterion, although this also included mosquitoes that had died. (**B**) Mortality was then assessed for all mosquitoes 24 h post-spray. Error bars indicate the standard deviation of the mean. Significance was determined using student t-test, *p < 0.05, ns, not significant. WT: the local wild strain of *Ae*. *aegypti*; LG: a susceptible strain of the laboratory-generated *Ae*. *aegypti* without drug resistance.

As shown in Fig 2, when the insecticide was diluted 400-fold (400x) we detected differences between WT and *w*AlbB-Tw in terms of 60 ppm delta-methrin and 275 ppm alpha-cypermethrin susceptibility (knockdown rate: 84.3 ± 3.2% vs. 100%, *p* = 0.013, 90.3 ± 3.7% vs. 100%, *p* = 0.02, Fig 2A; mortality rate: 93.3 ± 1.9% vs. 100%, *p* = 0.038, 95.6 ± 3.2% vs. 100%, *p* = 0.12, Fig 2B; student t-test). In *Wolbachia*-infected mosquitoes (*w*AlbB-Tw) and laboratory-produced *Ae*. *aegypti* (LG) exposed to 960 and 60 ppm of delta-methrin, 4,400 and 275 ppm of alpha-cypermethrin, 2,040 and 127.5 ppm of cyfluthrin, the mortality rate was consistently about 100%. While differences in response between the lines were detected for some insecticides (delta-methrin and alpha-cypermethrin), we observed no obvious effects related to insecticide resistance in mosquitoes infected with *Wolbachia*. Taken together, application of traditional insecticides may influence the effectiveness of *Wolbachia* release strategy.

### Fitness and mating behavior of *w*AlbB-Tw mosquitoes

One of the crucial concerns of programs focused on releasing mosquitoes carrying *Wolbachia* is fitness. The fitness of *w*AlbB-Tw mosquitoes (Generation 10; n = 40), quantified as the number of eggs laid, eggs hatched, and surviving adults, was compared with WT mosquitoes by statistical testing with the Mann-Whitney test. We found a slight significant difference in the number of eggs laid (*w*AlbB-Tw = 91.6 ± 11.7 and WT = 85.9 ± 12.9; *p* = 0.042, S3 Fig), but no differences in the number of eggs hatched (80.3 ± 10.1 and 81.6 ± 12.7; *p* = 0.18) or the number of surviving adults (76.7 ± 9.6 and 78.3 ± 11.6; *p* = 0.23). These results suggest that *w*AlbB-Tw females exhibited normal reproduction capacity.

**Fig 3:**
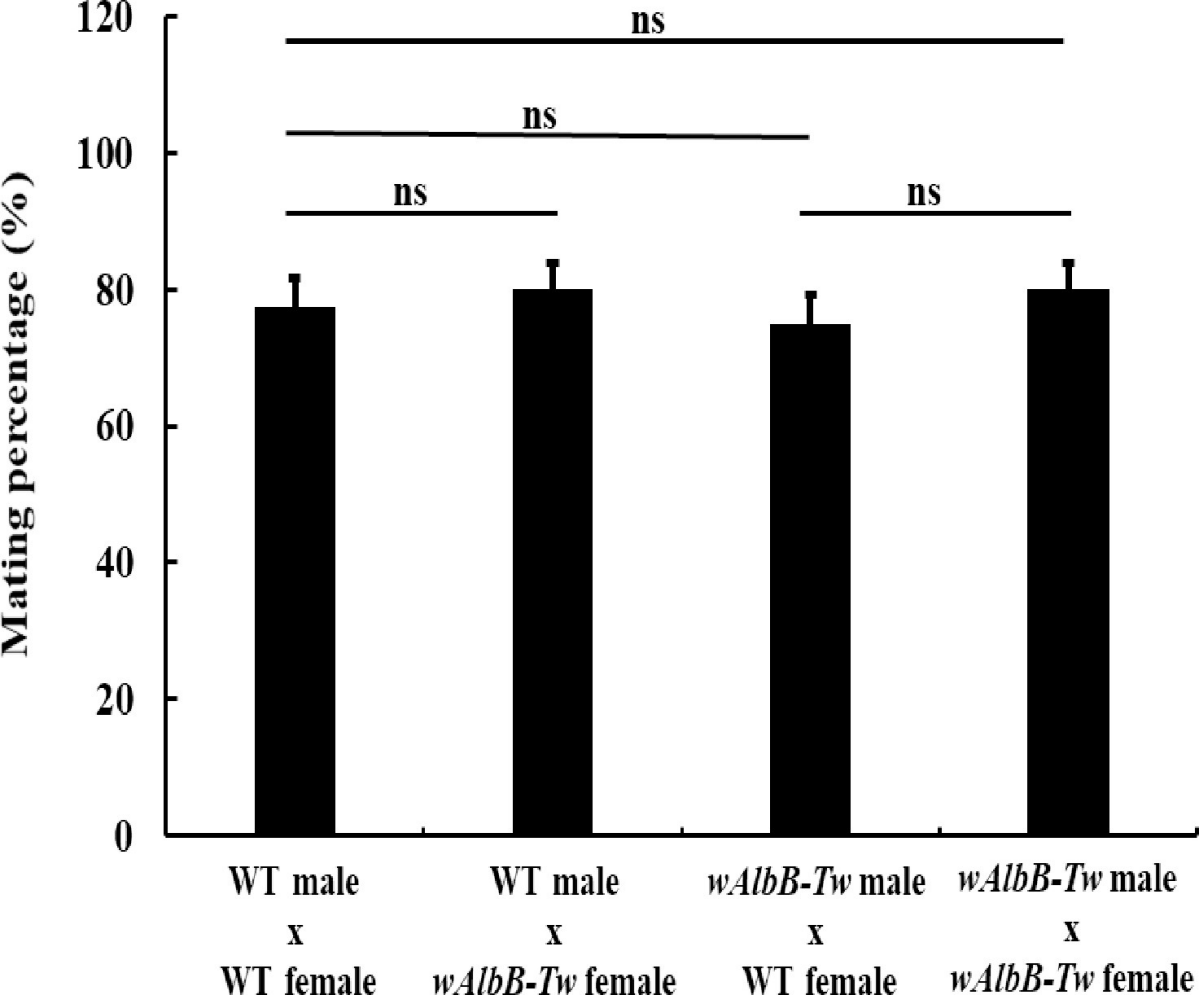
*w*AlbB-Tw male mating capacity analysis. The panel shows the statistical analysis of mating success or failure in the four mating situations. Data are represented as mean ± SD. The difference between the WT and *w*AlbB-Tw was evaluated using t-test and *p* value less than 0.05 was considered statistically significant. ns, not significant.

To determine whether *Wolbachia* influences male mating behavior activity, *w*AlbB-Tw males were allowed to mate with virgin females, with sperm presence in the female spermathecae used as a mating indicator (Fig 3). We found that the mosquito mating rate was approximately 75%–80% in the four reciprocal crosses, indicating no significant differences (compared with ♂WT x ♀WT, *p*>0.05). This suggests that the mating behavior ability of *w*AlbB-Tw mosquitoes is equivalent to that of wild type mosquitoes.

### Lifespan of *w*AlbB-Tw and wild type *Ae*. *aegypti*

**Fig 4:**
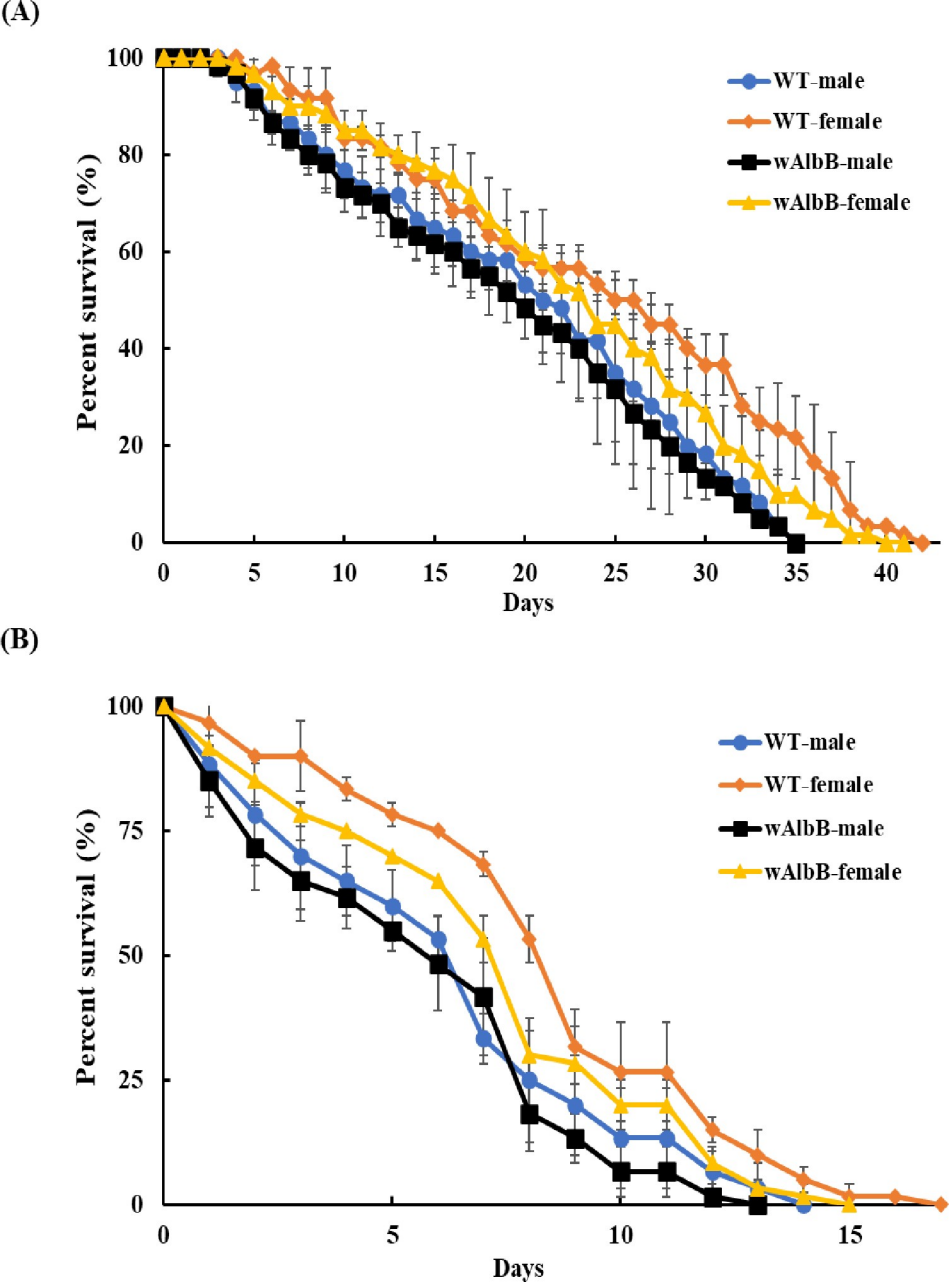
Comparing the lifespan of *w*AlbB-Tw and wild type (WT) *Ae*. *aegypti*. The lifespan of male and female mosquitoes in the *w*AlbB-Tw and WT strains were tested (**A**) in a laboratory environment (**B**) and semi-field simulation areas. All tests were started from 20 mosquitoes, and 10% sucrose was provided as daily food. The number of deceased mosquitoes were recorded, and the bodies were removed from the cage daily. Statistical analyses of lifespan were performed using the Log-Rank test. Plots combine three independent experiments and represent mean ± SD at each data point.

The lifespan of mosquitoes carrying *Wolbachia* is an important factor for managing the frequency of release in population suppression. For this purpose, we tested the lifespan of male and female *w*AlbB-Tw mosquitoes and compared it to the WT *Ae*. *aegypti* population. All lifespan tests started from 20 mosquitoes. In a controlled laboratory environment (Fig 4A), test results revealed no significant differences in lifespan between WT and *w*AlbB-Tw strains in both male (*p* = 0.83, 0.85, and 0.65; three replicates) and female (*p* = 0.37, 0.98, and 0.14; three replicates) when tested by the Log-Rank test.

In order to understand the lifespan in a natural environment, we also investigated WT and *w*AlbB-Tw strain lifespans in semi-field simulation areas (Fig 4B). Statistical analysis showed there was no significant difference between the lifespans of WT and *w*AlbB-Tw male mosquitoes (*p* = 0.38, 0.38, and 1; Log-Rank test). For female mosquitoes, we also found no significant difference in lifespan between WT and *w*AlbB-Tw strains (*p* = 0.29, 0.5 and 0.5; Log-Rank test). These results indicated that the lifespan of male *Ae*. *aegypti* was not affected by the *Wolbachia* infection.

### Suppression of wild type *Ae*. *aegypti* populations using *w*AlbB-Tw in laboratory trials

**Fig 5:**
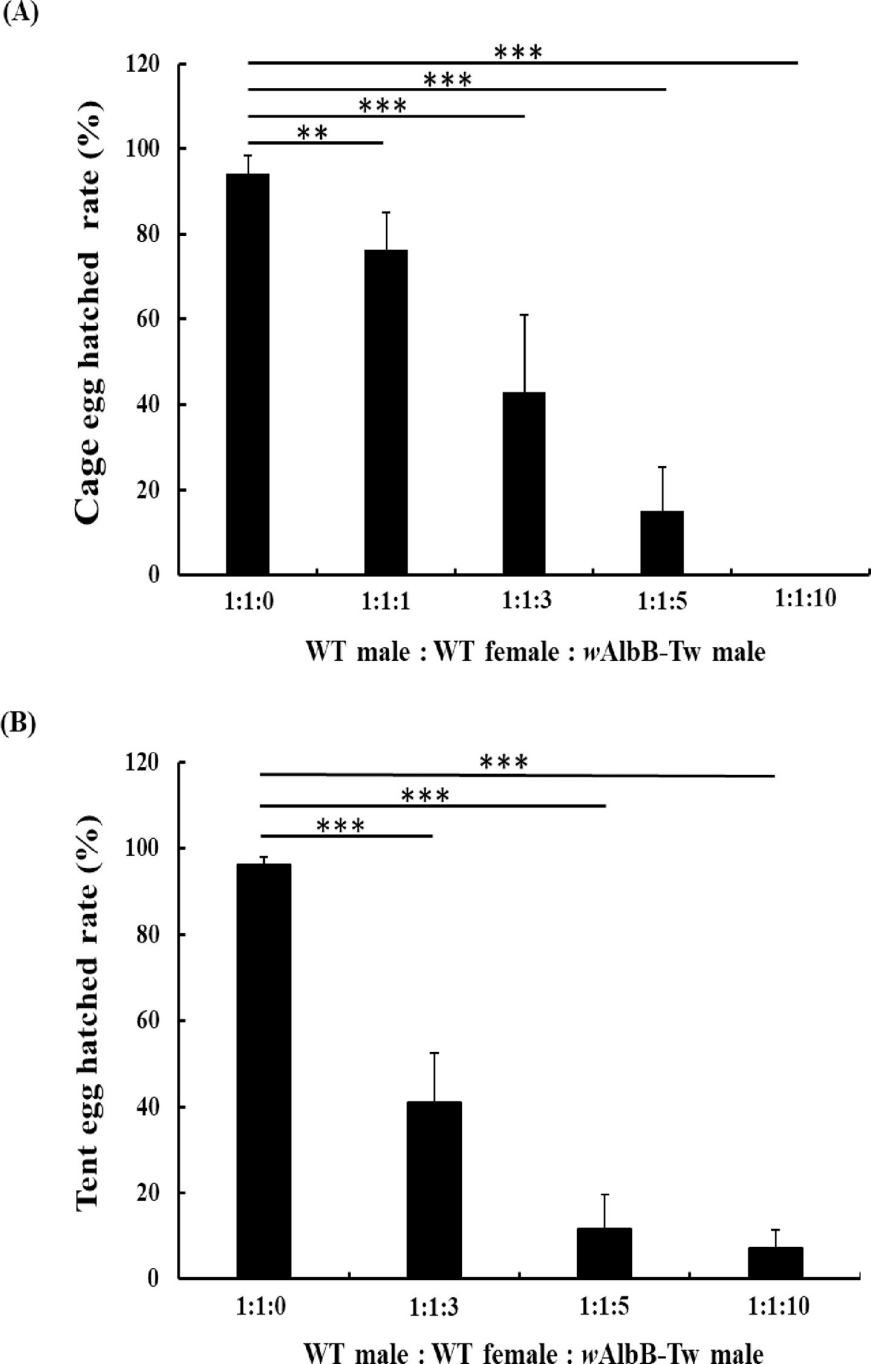
Suppression of wild type populations by release of *w*AlbB-Tw males under the laboratory condition. The (**A**) transparent acrylic cage (30 x 30 x 30 cm^3^) and (**B**) insect rearing tent (160 x 160 x 180 cm^3^, 150 mesh) populations contained different ratios of uninfected females, uninfected males, and *w*AlbB-Tw males. The levels of population suppression are expressed as the percentage of eggs hatched in each treatment. Each result represents the mean ± SD of five independent replicates. Significant *p* values are indicated by asterisks: *p < 0.05, **p < 0.01, ***p < 0.001.

To determine whether the CI expressed by *w*AlbB-Tw lines could be used for population suppression, we tested transparent acrylic cage and insect rearing tent populations containing different ratios of untransinfected males, untransinfected females, and transinfected males. In the laboratory transparent acrylic cages, WT mosquito populations were suppressed in a ratio-dependent manner with *w*AlbB-Tw males. At mating ratios of ♂WT and virgin ♀WT vs ♂*w*AlbB-Tw were 1:1:1, 1:1:3, 1:1:5 and 1:1:10, viable eggs were still observed (egg hatch rate [1:1:1] = 76.28 ± 8.9%, *p < 0*.*001*; egg hatch rate [1:1:3] = 42.9 ± 18.1%, *p < 0*.*001*; egg hatch rate [1:1:5] = 14.9 ± 10.5%, *p < 0*.*001*) but a significant reduction in the egg hatch rate was detected at mating ratios of 1:1:10 (egg hatch rate [1:1:10] = 0.0 ± 0.0%) (S2 Table). Under these conditions, population suppression reached 100% for males released at the ratio of 1:1:10 (Fig 5A). Similar results were obtained in the insect rearing tent experiments; higher over flooding ratios can significantly reduce egg hatching rates and enhance the population suppression effect (mean egg hatch rate [control group 1:1:0] = 96.2 ± 1.9%, *p < 0*.*001*, mean egg hatch rate [1:1:10] = 7.2 ± 4.3%, *p < 0*.*001*, S3 Table and Fig 5B). These data clearly demonstrate that *w*AlbB-Tw mosquitoes can be used for population suppression by inducing CI.

### Evaluating *w*AlbB-Tw mosquito mating competitiveness in semi-field conditions

**Fig 6:**
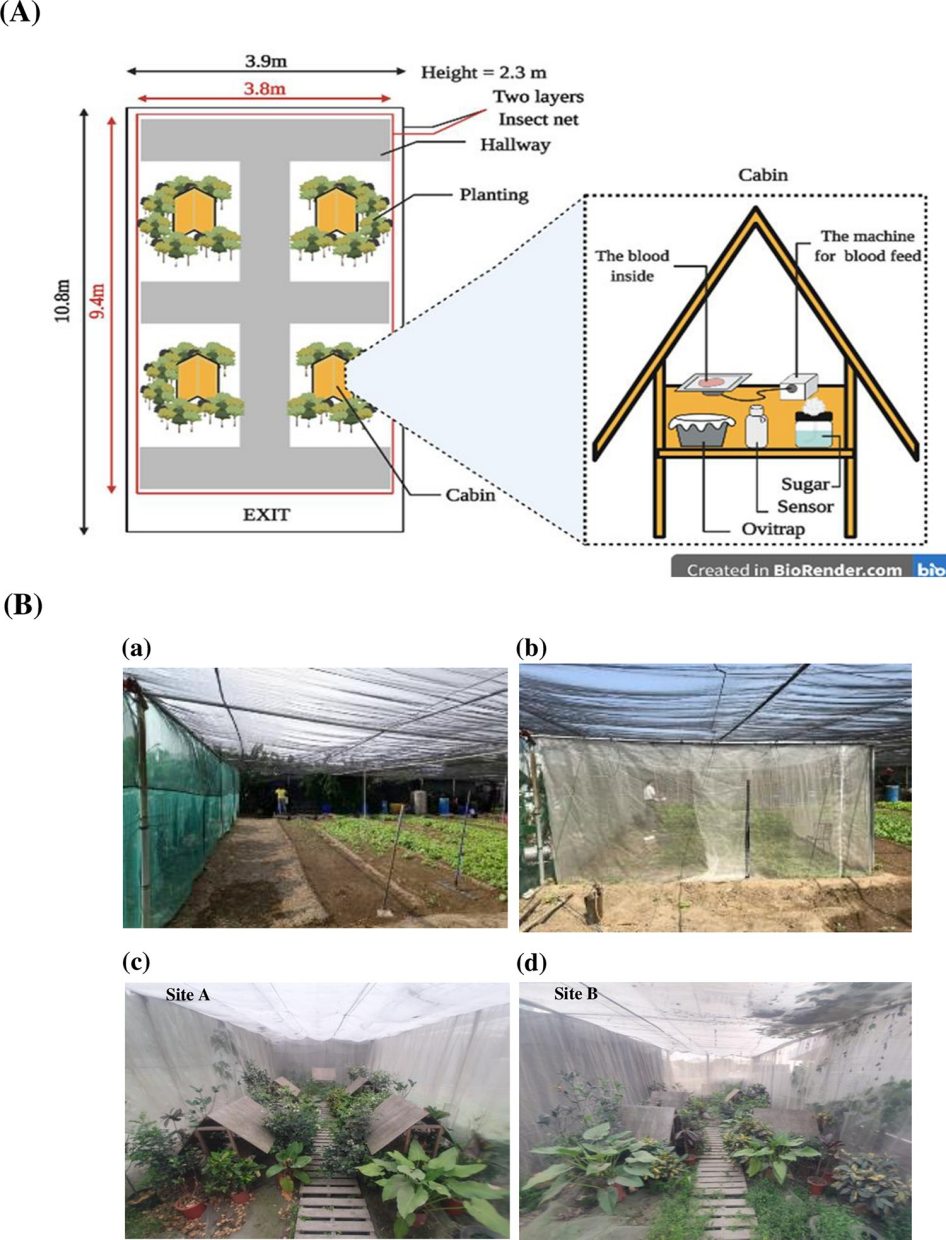
Layout drawing of the semi-field building simulation area. (**A**) Inside the building, four wooden cabin structures simulating a house were situated at each experiment site as a shelter for mosquitoes. Sensors located in the test site collected data on ambient temperature and relative humidity, which also contained ovitraps and artificial membrane feeder system inside the field. The figure is created via BioRender.com. (**B**) (a, b) The initial appearance of the semi-field building and (c, d) the simulated courtyard in these areas. The remainder of the internal site was covered with mulch and potted plants to emulate a typical yard.

The semi-field simulation test is an important step prior to the implementation of population suppression release efforts in the field. We tested the potential for *w*AlbB-Tw strains to suppress mosquito populations in the semi-field building system (Fig 6A and 6B). We also monitored temperature and humidity of the test site throughout the experiments. During the trial, we did not detect differences in temperature between test sites (site A 17.5 to 29.8°C and site B 18.1 to 30.7°C; median temperature site A = 25.4°C, B = 26.1°C). During the same period, RH ranged from 77.6% to 99.9% in site A and 79.4% to 100% in site B. Median RH was slightly higher in site B (90.0%) than site A (88.0%). However, in the temperature and RH analysis, this difference was not statistically significant (Mann-Whitney test, *p*> 0.05) (S4A and S4B Fig). At the same time, analysis of the accumulated rainfall (S4C Fig) also showed no obvious differences.

**Fig 7:**
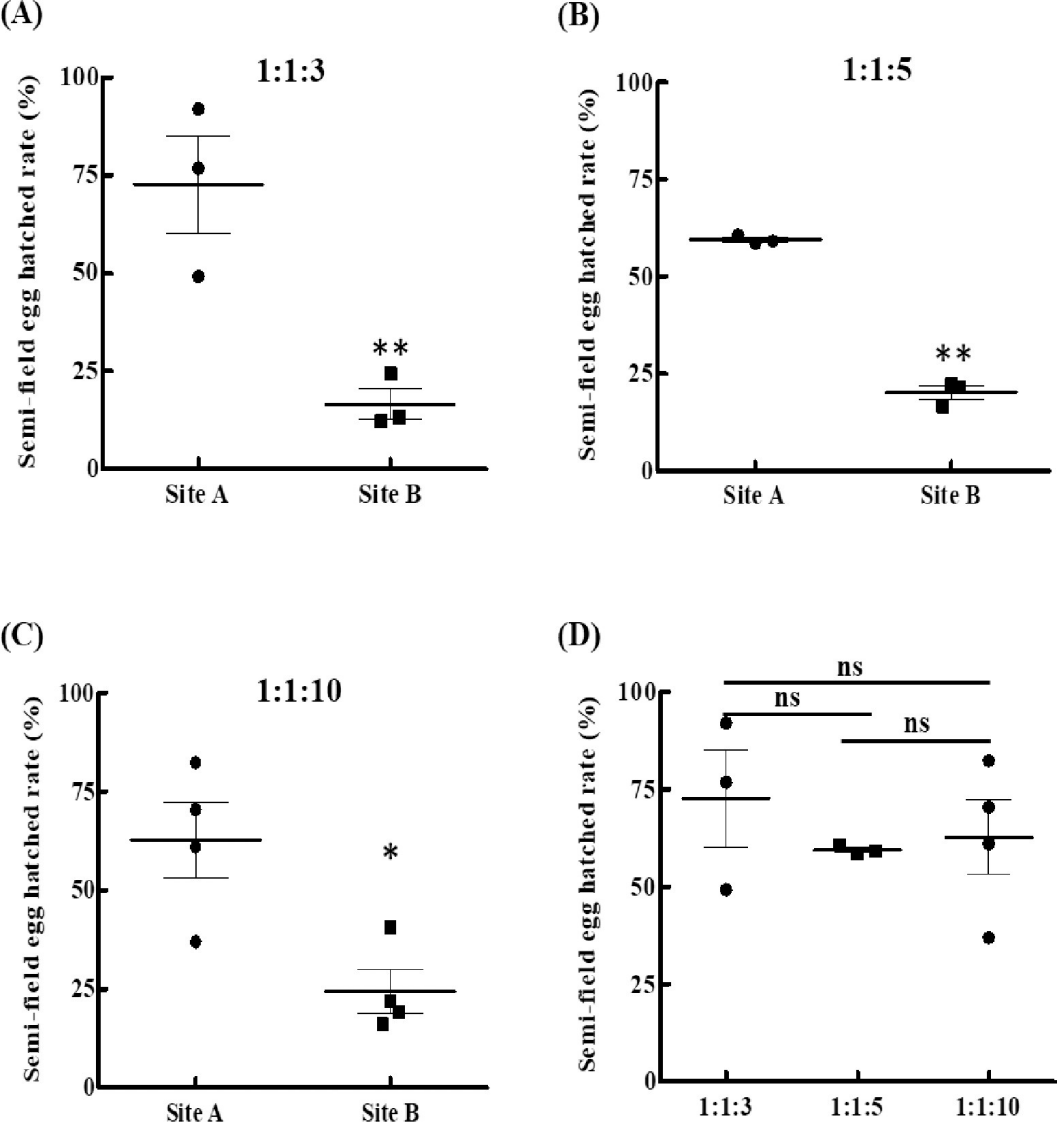
Suppression of wild type populations by release of *w*AlbB-Tw males under the semi-field condition. The population was tested under different ratios of uninfected females, uninfected males, and *w*AlbB-Tw males in (**A**) 1:1:3, (**B**) 1:1:5, and (**C**) 1:1:10. Population suppression is expressed as the percentage of eggs hatched and followed the procedure described in the Materials and Methods. Each result represents the mean ± SD of four independent measurements. (**D**) Analysis of the hatch rate at different environmental conditions (temperature and humidity) in the control group (Site A). Significance was determined using student t-test, *p < 0.05, **p < 0.01, ns, not significant.

To evaluate *Wolbachia*-transfected mating competitiveness in the semi-field location, three releasing ratios (female WT: male WT: male *w*AlbB-Tw in 1:1:3, 1:1:5, and 1:1:10) were tested in our study. The semi-field experiments were performed from 2019/04/12–2020/06/10. From 2019/04/12–2019/07/02, the field environment temperature and humidity were 27.90 ± 2.31°C, 87.23 ± 3.71% and 27.80 ± 2.20°C, 86.70 ± 2.86% in Site A and Site B, respectively. During this period, the hatch rate of 1:1:0 group and 1:1:3 group was 72.70 ± 21.66% and 16.57 ± 6.80%, respectively. The hatch rate was significantly decreased in the field containing male *w*AlbB-Tw (student t-test; *p* = 0.004, Fig 7A). From 2019/08/16–2019/11/21, the field environment temperature and humidity were 26.27 ± 0.93°C, 87.87 ± 9.94% and 26.33 ± 1.02°C, 85.70 ± 8.29% in Site A and Site B, respectively. During this period, the hatch rate of 1:1:0 group and 1:1:5 group was 59.48 ± 1.12% and 20.12 ± 3.07%, respectively. The hatch rate also was significantly decreased in the field containing male *w*AlbB-Tw (student t-test; *p* = 0.007, Fig 7B).

From 2020/01/17–2020/06/10, the field environment temperature and humidity were 22.53 ± 3.60°C, 84.65 ± 5.37% and 22.70 ± 3.55°C, 84.75 ± 6.21% in Site A and Site B, respectively. During this period, the hatch rate of 1:1:0 group and 1:1:10 group was 62.73 ± 19.25% and 24.45 ± 11.02%, respectively. The hatch rate was significantly decreased in the field containing male *w*AlbB-Tw (student t-test; *p* = 0.02, Fig 7C). According to the above results, the hatch rate was significantly decreased after male *w*AlbB-Tw competed for mating with male WT in semi-field experiments. The temperature and humidity of Site A and Site B were similar, and the hatch rate in the control group (Site A) was 72.70 ± 21.66%, 59.48 ± 1.12%, and 62.73 ± 19.25%, with no statistical difference between these data points (Fig 7D).

At the same time, we also analyzed the results of each mating competitiveness during the test period (S4 Fig and S4 Table), and found that in our test, the temperature and humidity of the test site did not seem to affect the population suppression results of the semi-field. These results suggest that the hatch rate does not fluctuate over time with varying environmental conditions (temperature and humidity) in our test. Together, our semi-field experiments provide scientific evidence that the *Wolbachia* strategy is feasible to prevent dengue virus infection in Taiwan.

## Discussion

Mosquito-borne viruses continue to cause major public health problems worldwide. *Wolbachia* as a novel vector control tool is gaining global attention and support [11,16,49]. There are two strategies for using *Wolbachia* in vector-borne disease control: (1) population suppression by only releasing male mosquitoes, which reduces the population density of field mosquito vectors and decreases disease transmission; and (2) population replacement by releasing males and females together to replace field mosquito vector populations with *Wolbachia-*transinfected populations to inhibit virus spread. The purpose of our study was to conduct a pilot trial for suppressing the local strain of *Ae*. *aegypti* using *Wolbachia* to produce sterile males in our semi-field system. This includes the complete characterization of the *Wolbachia*-infected local strain. Our results indicate that under semi-field conditions the *w*AlbB-Tw strain has the ability to reduce *Ae*. *aegypti* populations by more than 70%.

For the population suppression strategy to be successful, the number and ratio of released male mosquitoes carrying *Wolbachia* is critical, primarily due to the need for sufficient *Wolbachia-*transinfected males to compete with wild males. Release frequency, distribution of wild populations, and longevity of released males also affect this release ratio. Therefore, selecting the correct release ratio is an important factor for control strategy success. Adjusting the number of males released according to environmental or climatic factors is also critical. For example, during the rainy season, mosquito populations usually increase. It may be helpful to increase the number of males released a few weeks after heavy rain to increase the reproductive competitive advantage of *Wolbachia-*transinfected males in the wild [37]. Furthermore, before release, appropriate control measures should be considered to reduce initial *Aedes* mosquito populations, thereby increasing suppression efficiency.

Our results indicated that the presence of *Wolbachia* had only a minimal effect on host fitness, which may be related to the background host genome and the *Wolbachia* strain. Because the *Ae*. *aegypti* strains infected with *w*AlbB have been backcrossed at least ten times, their genetic backgrounds are highly similar to those of the natural wild strains from Tainan, Taiwan. The fitness effect of this infection is important for understanding the spread and maintenance of *Wolbachia* within and among host populations. However, a full elucidation of fitness effects requires careful control of the host genetic background [50–52]. Another report has highlighted that *w*AlbB infection generates strong CI and perfect maternal transmission whilst having minimal effects on host fitness in terms of fecundity and egg hatch, similar to *w*Mel [41]. Our study also suggests that *w*AlbB infections had minimal effects on the fitness of the *Ae*. *aegypti* studied.

Under laboratory conditions, we determined the optimal release ratio of sterile *Wolbachia*-transinfected male mosquitoes to be 10:1 for *w*AlbB-Tw males to compete with wild type males and introduce complete or nearly complete sterility in wild type females. This is similar to a previous study investigating the use of radiation exposure on *Ae*. *albopictus* to develop sterile mosquitoes, which determined that a sterile-to-wild male ratio above 5:1 was necessary to ensure an efficient sterile male release [53]. In our semi-field experiment, a 3:1 ratio was sufficient to reach 70% suppression, although this suppression rate did not increase with ratios of 5:1 or 10:1 (Fig 7). This suggests that a 3:1 ratio will be a suitable starting point for future release trials. In addition, the mark-recapture method should be used to estimate the size of the wild-type population to predict the number of *Ae*. *aegypti* carrying *Wolbachia* that should be released [54–56]. This is a key parameter for implementing a vector control plan based on a male release strategy. In semi-field trials, too high a density of male *Wolbachia* mosquitoes near the ovitrap attempting to mate with females coming to lay eggs may have interfered with egg laying and the timing of oviposition, reducing the overall number of eggs laid in a manner unrelated to *Wolbachia* suppression. A balanced release ratio also needs to consider the feedback from residents as well as the environmental temperature during trial. During our testing of ratios of ♂WT vs ♂*w*AlbB-Tw 1:5 and 1:10, 12 days of severe cold weather may have affected mosquito lifespan, motility and fertility. These closed experiments perform under the real weather conditions provide a good basis for an open release program under semi-field and almost natural conditions.

Previous studies have shown that *Aedes* spp. are the most widely distributed global mosquito species, and genetic structure variations affect biological control strategies [57,58]. Genetic structure variations may also show different *Wolbachia* infection rates, as found in *Ae*. *albopictus* subspecies in China [59]. In addition, Garcia et al. indicated that deployed *Wolbachia*-transinfected mosquitoes should match the insecticide resistance of wild populations to achieve effective epidemic prevention [60]. As shown in Fig 2, we found different responses to some insecticides between mosquito lines, suggesting genetic variation. Using high concentrations of insecticides, there were no obvious differences in knockdown rate and mortality between *w*AlbB-Tw and uninfected strains (WT). Using low concentrations treatments can however cause differences to appear, with *w*AlbB-Tw seemingly not resistant to insecticides (Fig 2). These results suggest that *Wolbachia* transinfection should not influence the chemical control of *Ae*. *aegypti*. However, the current epidemic prevention policy still relies on insecticides to target mosquito populations. This is unfavorable for *w*AlbB-Tw, which is not resistant. In order for Taiwan to implement a large-scale release of *w*AlbB-Tw mosquitoes in the future, the spraying of insecticides in the release area may need to be at least partially restricted so that *Wolbachia* can effectively show long-term effects of population suppression. Alternatively, every third generation may need to be mated with local insecticide-resistant wild type *Ae*. *aegypti* mosquitoes to increase gene flow and refresh the genetic pool between populations, thus leading to insecticide-resistant *Wolbachia*-transinfected mosquitoes. Only then can there be effective population prevention and control [61]. Genetic variation among populations is therefore another important research topic for arthropod-borne virus infection and disease transmission, and further study will help outline biological control plans that benefit from matched genetic profiles of released and local populations.

Although the semi-filed facilities used here had some major advantages (such as a relatively low cost), there were still some limitations. Due to limitations of the construction area, we were constricted to two test sites only for conducting multiple sequential experiments (Fig 6). This is further complicated due to seasonal differences between consecutive runs. Although the relative humidity between the two cages is slightly different, these differences are less than 5%. We do not consider these to be biological barriers for field trials, but these environmental parameters should be listed as important references. The limited space in the facilities may also greatly restrict flight activities. Further, as the use of pesticides must be avoided, the invasion of predators may be a problem that affects the mortality of the target species. Therefore, the results of semi-field experiments must be interpreted carefully, especially when extrapolating to field conditions.

Each of the two *Wolbachia* control strategies has their own advantages and disadvantages. There are differences in cost effectiveness, for example, as male only releases require the separation of male and female pupae prior to eclosion, which consumes large amounts of manpower and material resources. On the other hand, the cost is relatively small in the replacement method. In terms of the release schedule, the suppression control option also requires continuous release of male mosquitoes to effectively suppress mosquito density. In replacement releases, the release time can be shorter than that required for population suppression. Here, there does not need to be a long, continuous release to effectively prevent the spread of dengue fever. However, releasing only male mosquitoes carrying *Wolbachia* would be more acceptable from a community perspective because male mosquitoes do not suck blood and can gradually reduce the number of mosquitoes in the wild. This is not the case for the replacement strategy, as female and male mosquitoes carrying *Wolbachia* are released together. Here, female mosquitoes sucking human blood are less acceptable to the public, so it is difficult to promote and communicate compared to a population suppression strategy.

Recently, the use of new vector control strategies to combat arthropod-borne virus transmission has been widely discussed, and research has included artificial infection with *Wolbachia* to reduce population size and vector capacity as well as genetic engineering methods under RNAi based genes [62–64] and neutralizing antibodies [65]. Further, *Wolbachia* infection may reveal maternal transmission leakage and incomplete CI, which will increase the proportion of infected mosquitoes required for release and reduce invasion speed. Despite this, infection with *Wolbachia* is still considered an environmentally friendly strategy for controlling arthropod-borne diseases. The use of genetically modified (GM) mosquitoes instead of mosquitoes carrying *Wolbachia*, there are biological safety considerations and strict implementation requirements.

Taiwan is located in a subtropical region, and the high temperature and humid environment is conducive to the breeding and reproduction of mosquitoes. Particularly in southern Taiwan, vector-borne diseases can be seen throughout the year. Dengue fever is the most important vector-borne infectious disease in summer in Taiwan, and it is also a key prevention and control project of the government’s health and environmental protection unit. In order to effectively control the density of mosquito populations, government institutions rely on chemical prevention and treatment. In addition to the impact on the environment and people’s health, this reliance gradually results in the accumulation of drug resistance in mosquito populations, with ever greater concentrations of insecticides being used to achieve the same control effect. Such a vicious circle will deepen the difficulty of disease control. Therefore, we aim to promote the use of *Wolbachia* as a method to control vector mosquitoes in Taiwan by effectively reducing the density of *Ae*. *aegypti* and thus the spread and incidence of dengue fever. In the future population suppression plan, the current program will select a suitable release area in southern Taiwan as a pilot experimental area. The program will also conduct a mosquito density survey and make a promotional video to facilitate community communication. We envision that the results of the semi-field experiment and subsequent planning can be used as a guide for Taiwan’s future population suppression, thereby reducing the threat of dengue fever. The implementation of such innovative strategies to prevent and treat vector diseases in Taiwan will have a major and far-reaching impact on public health.

*Wolbachia*-based DENV control strategies show great potential as a sustainable, economic, and environmentally friendly solution compared to other methods. Therefore, *Wolbachia* could be implemented as a biological control tool to combat mosquito vectors and vector-borne diseases, especially in remote areas, difficult-to-access areas, and areas where existing interventions cannot eliminate disease transmission. However, further research is needed to improve upon current knowledge of emerging vector-borne diseases and build a foundation for developing innovative population control tools.

## Supporting information

S1 Fig Presence of *Wolbachia* in *w*AlbB-Tw mosquitoes’ midgut and ovary. Immunofluorescence assay showing distribution of *Wolbachia* (green) in the (**A**) midgut and (**B**) ovary of transinfected *Ae*. *aegypti* female mosquitoes. DNA is stained with DAPI (blue) and the cytoskeleton is stained with phalloidin (red). Scale bar: 50 μm. WT: Tainan local wild type.
(TIF)

S2 Fig Inhibition of dengue virus (DENV) infection in the *w*AlbB-Tw. Mosquito midguts, salivary glands and the saliva were collected at 7- and 14-days post blood meal (PBM) containing DENV, and the virus titer was determined by plaque assays using BHK21 cells. We performed the virus challenges using (**A**) DENV-1 of the Myanmar strain, (**B**) DENV-2 of the NGC strain, (**C**) DENV-3 of the 98TW503 strain, and (**D**) DENV-4 of the H241 strain on the *w*AlbB-Tw and Tainan local wild type (WT). Saliva samples were collected after 30 min in a P200 tip containing 5 μL of FBS and then expelled into 45 μL of L-15 media for analysis. For each mosquito (n = 30), the mean viral titer is plotted, and the standard error of the mean is indicated. Uninfected mosquito samples were not plotted or included when determining the mean or standard error of the mean. The infection rates (IR) efficiency of the mosquito lines are indicated as percentages and shown at the bottom of the chart. The Mann–Whitney rank sum test was used to analyze the difference in the virus titers. Significant *p* values are indicated by asterisks: *p < 0.05, **p < 0.01.
(TIF)

S3 Fig *w*AlbB-Tw female mosquito reproduction capacity assay. Reproduction capacity of *w*AlbB-Tw (Generation 10, n = 40) and WT (Tainan city local field *Ae*. *aegypti* strain) measured by the numbers of laid eggs, hatched eggs, larvae and survived adult. Data are represented as mean ± SD. The difference between the WT and *w*AlbB-Tw was evaluated using Mann-Whitney test and *p* value less than 0.05 was considered statistically significant. *p < 0.05, ns, not significant.
(TIF)

S4 Fig Collection of environmental parameters in the semi-field building simulation area. The sensors located at the test site recorded data monitoring ambient temperature and relative humidity once an hour. The values represent the average data collected in each experiment **(A** and **B).** We also concurrently collected accumulated rainfall data during each test period **(C).**
(TIF)

S1 Table DENV replication reduced in wAlbB-Tw *Ae. aegypti*. (TIF)

S2 Table Mating competitiveness in transparent acrylic cage. (TIF)

S3 Table Mating competitiveness in insect rearing tent. (TIF)

S4 Table Competition cages with different ratios of *w*AlbB-Tw males showing the number of hatched eggs against the total number of eggs collected for each time point. (TIF)
