# Supplementary figures and images for: Lab-scale characterization and semi-field trials of Wolbachia Strain wAlbB in a Taiwan Wolbachia introgressed Ae. aegypti strain
Source: PLoS Negl Trop Dis. 2022 Jan 11;16(1):e0010084. doi: 10.1371/journal.pntd.0010084 (PMC8752028; doi:10.1371/journal.pntd.0010084)

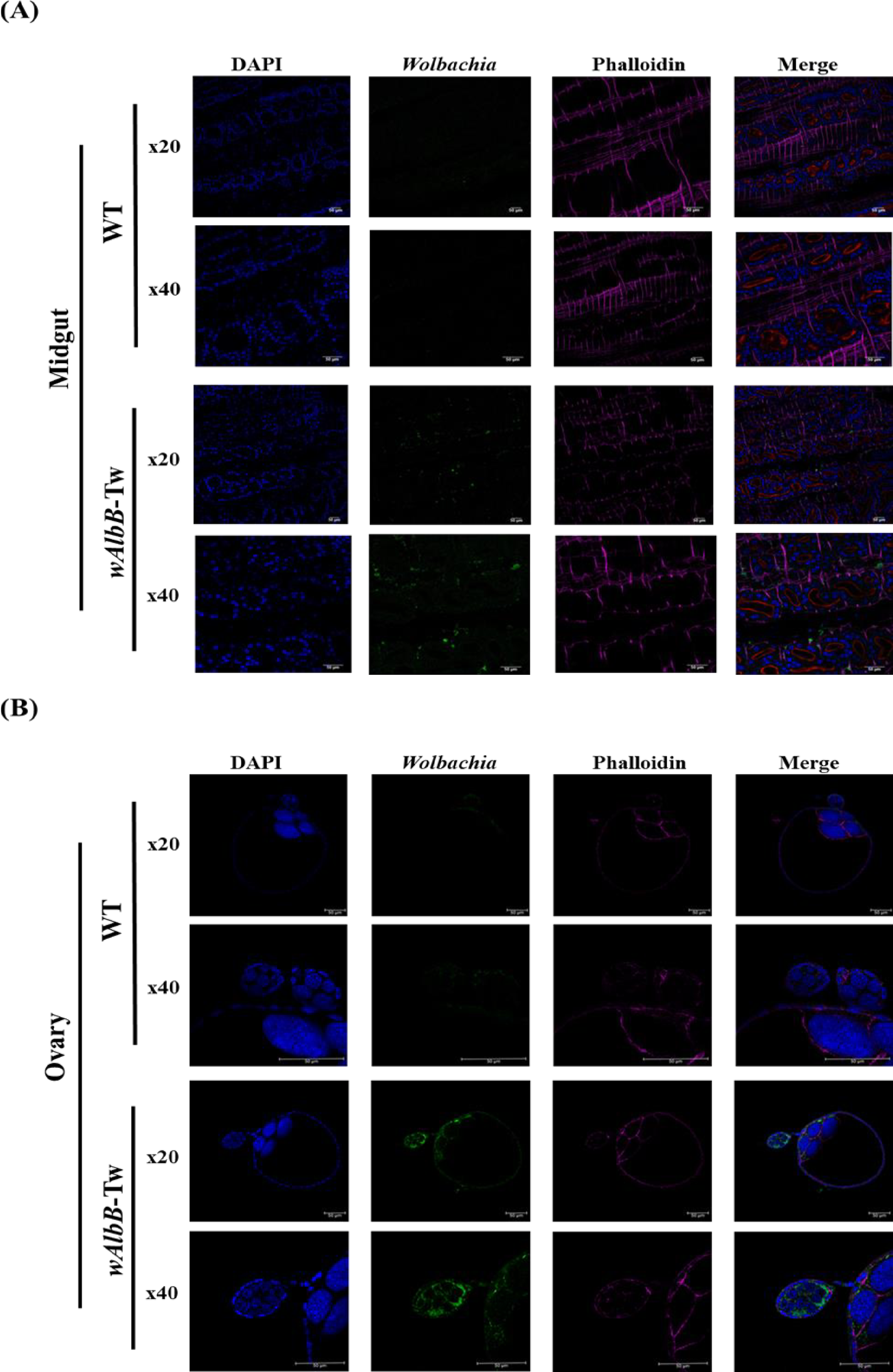

Supplement: S1 Fig — Immunofluorescence assay showing distribution of Wolbachia (green) in the (A) midgut and (B) ovary of transinfected Ae. aegypti female mosquitoes. DNA is stained with DAPI (blue) and the cytoskeleton is stained with phalloidin (red). Scale bar: 50 μm. WT: Tainan local wild type. (TIF) [file pntd.0010084.s001.tif]

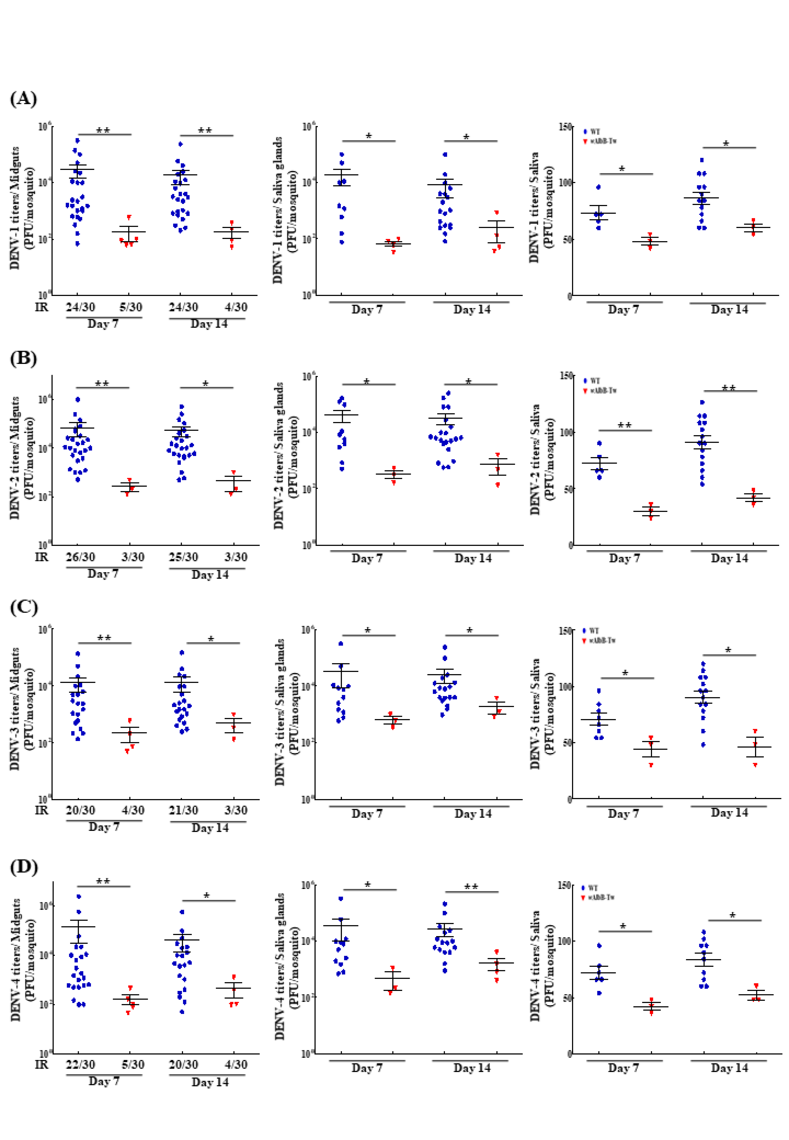

Supplement: S2 Fig — Mosquito midguts, salivary glands and the saliva were collected at 7- and 14-days post blood meal (PBM) containing DENV, and the virus titer was determined by plaque assays using BHK21 cells. We performed the virus challenges using (A) DENV-1 of the Myanmar strain, (B) DENV-2 of the NGC strain, (C) DENV-3 of the 98TW503 strain, and (D) DENV-4 of the H241 strain on the wAlbB-Tw and Tainan local wild type (WT). Saliva samples were collected after 30 min in a P200 tip containing 5 μL of FBS and then expelled into 45 μL of L-15 media for analysis. For each mosquito (n = 30), the mean viral titer is plotted, and the standard error of the mean is indicated. Uninfected mosquito samples were not plotted or included when determining the mean or standard error of the mean. The infection rates (IR) efficiency of the mosquito lines are indicated as percentages and shown at the bottom of the chart. The Mann–Whitney rank sum test was used to analyze the difference in the virus titers. Significant p values are indicated by asterisks: *p < 0.05, **p < 0.01. (TIF) [file pntd.0010084.s002.tif]

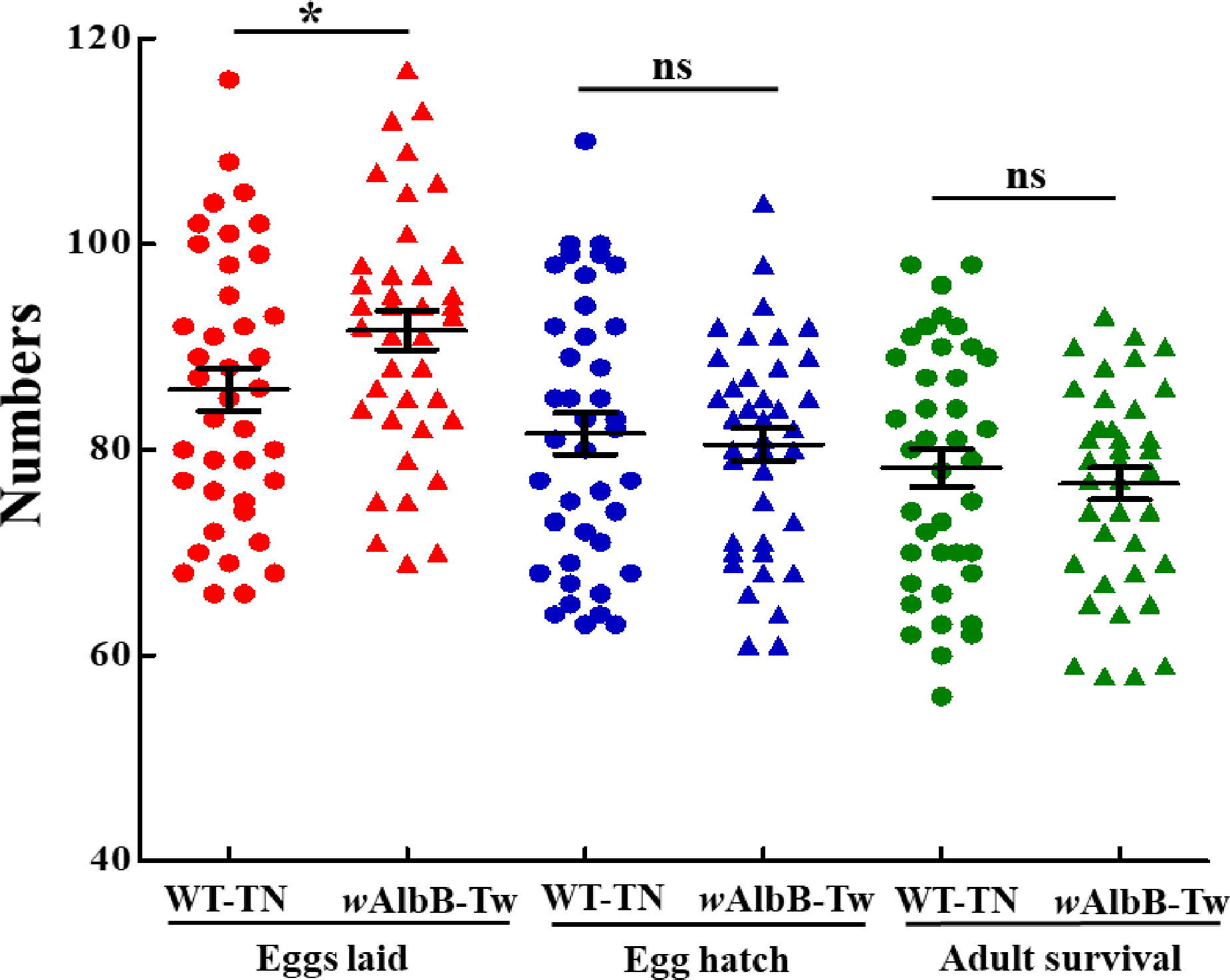

Supplement: S3 Fig — Reproduction capacity of wAlbB-Tw (Generation 10, n = 40) and WT (Tainan city local field Ae. aegypti strain) measured by the numbers of laid eggs, hatched eggs, larvae and survived adult. Data are represented as mean ± SD. The difference between the WT and wAlbB-Tw was evaluated using Mann-Whitney test and p value less than 0.05 was considered statistically significant. *p < 0.05, ns, not significant. (TIF) [file pntd.0010084.s003.tif]

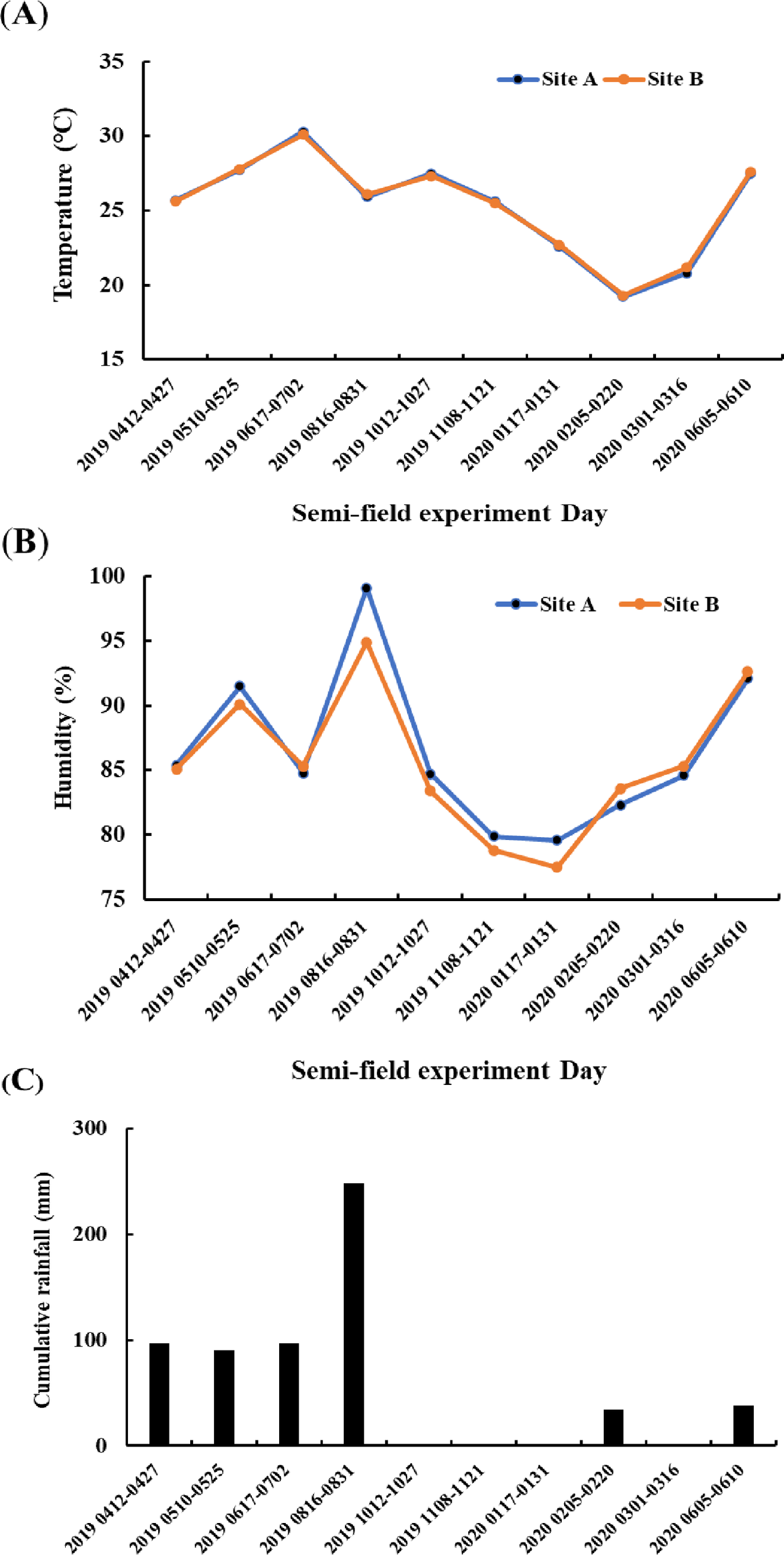

Supplement: S4 Fig — The sensors located at the test site recorded data monitoring ambient temperature and relative humidity once an hour. The values represent the average data collected in each experiment (A and B). We also concurrently collected accumulated rainfall data during each test period (C). (TIF) [file pntd.0010084.s004.tif]

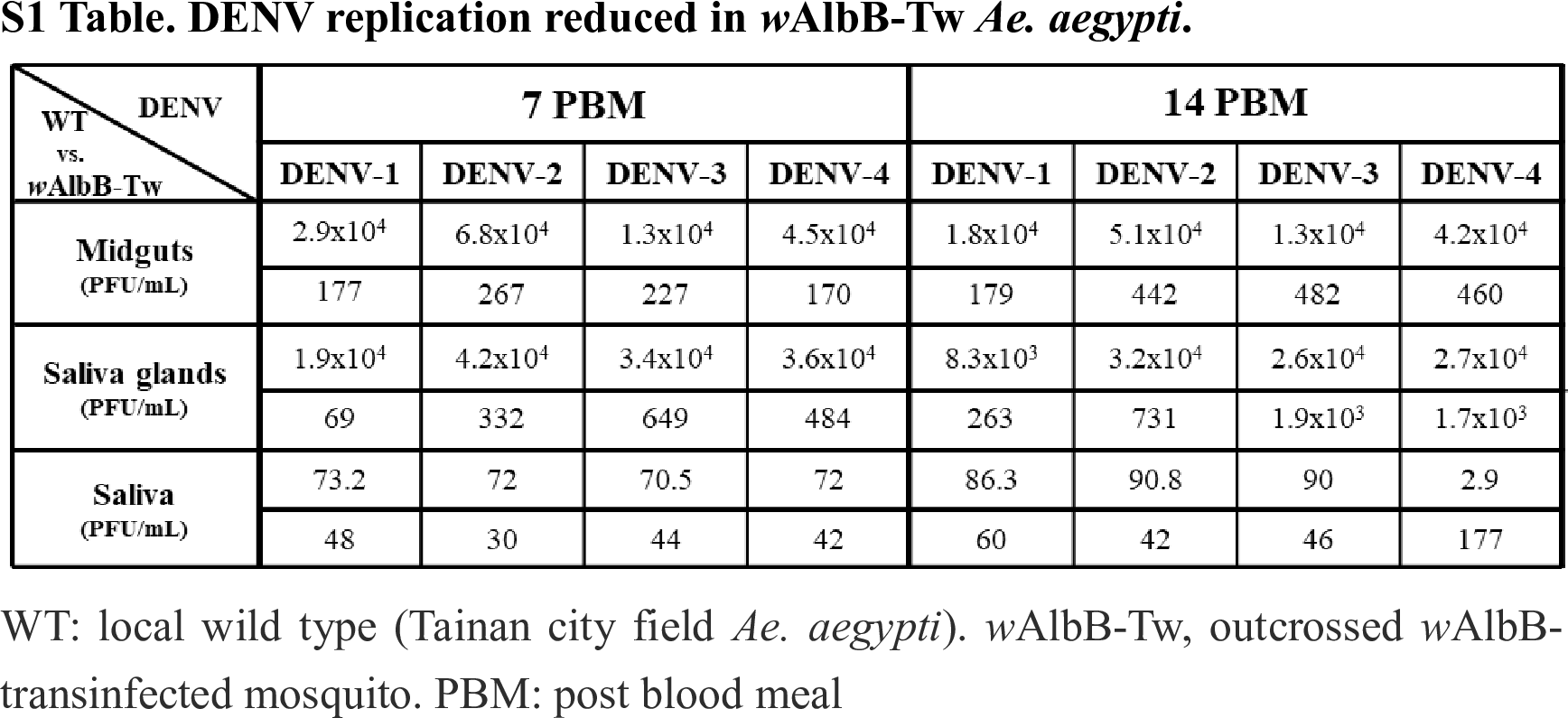

Supplement: S1 Table — (TIF) [file pntd.0010084.s005.tif]

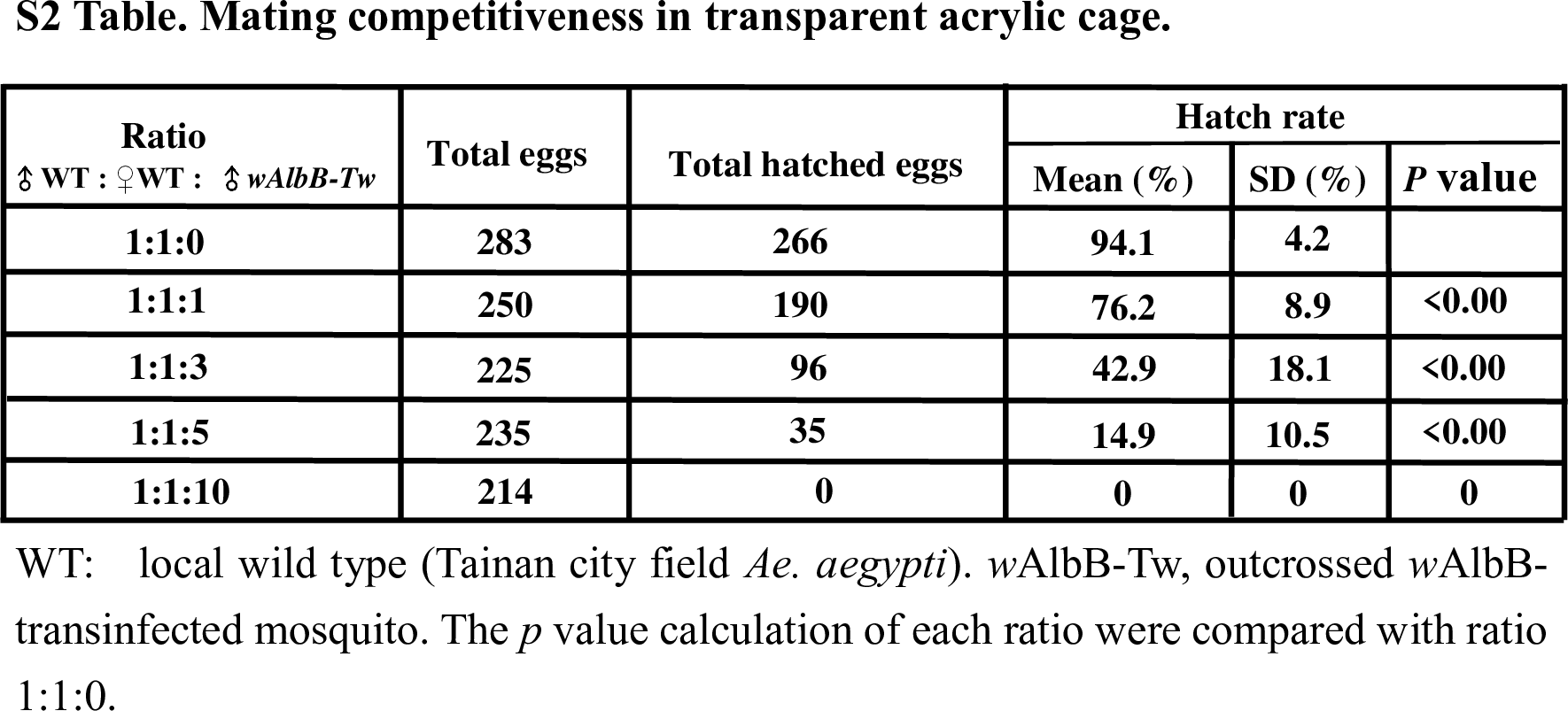

Supplement: S2 Table — (TIF) [file pntd.0010084.s006.tif]

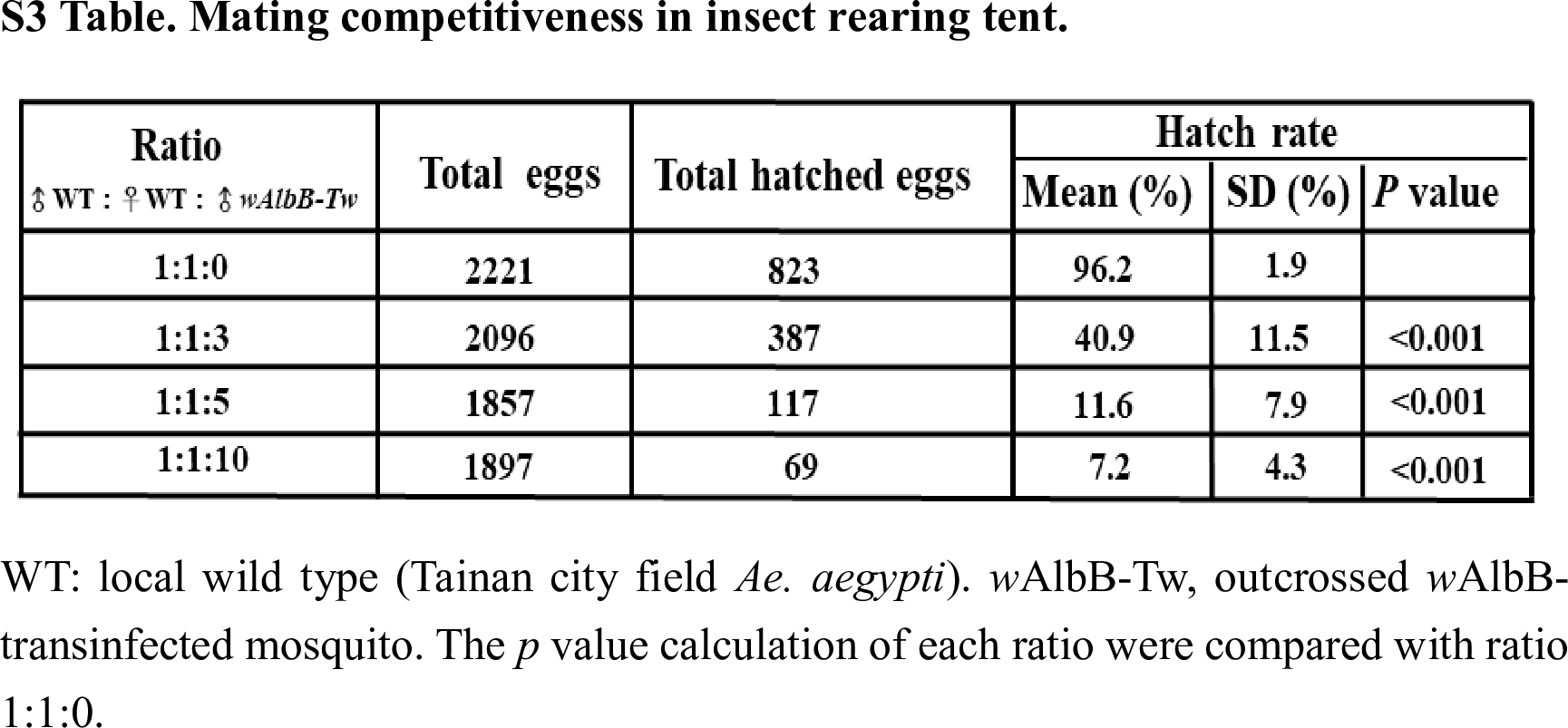

Supplement: S3 Table — (TIF) [file pntd.0010084.s007.tif]

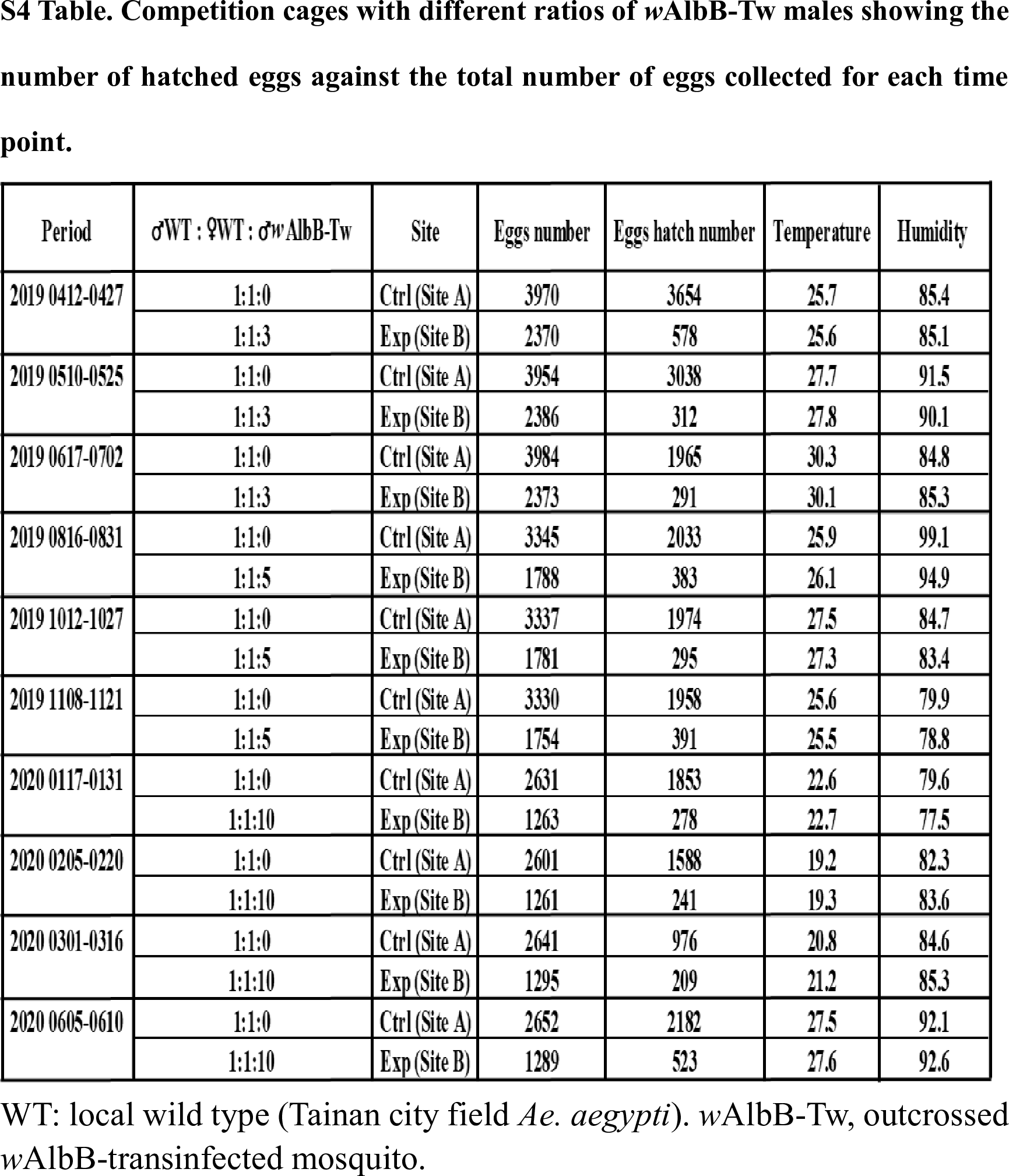

Supplement: S4 Table — (TIF) [file pntd.0010084.s008.tif]
